# Optimized Extraction of Total Triterpenoids from Jujube (*Ziziphus jujuba* Mill.) and Comprehensive Analysis of Triterpenic Acids in Different Cultivars

**DOI:** 10.3390/plants9040412

**Published:** 2020-03-27

**Authors:** Lijun Song, Li Zhang, Long Xu, Yunjian Ma, Weishuai Lian, Yongguo Liu, Yonghua Wang

**Affiliations:** 1 School of Food Science and Engineering, South China University of Technology, Guangzhou, Guangdong 510641, China; 201710104471@mail.scut.edu.cn (L.S.); 201610104342@mail.scut.edu.cn (L.X.); femayj@mail.scut.edu.cn (Y.M.); 201410104818@mail.scut.edu.cn (W.L.); 2 College of Life Science, Tarim University, Alar, Xinjiang 843300, China; 120100039@taru.edu.cn; 3 Beijing Advanced Innovation Centre for Food Nutrition and Human Health, Beijing Key Laboratory of Flavor Chemistry, Beijing Technology and Business University, Beijing 100048, China; liuyg@th.btbu.edu.cn

**Keywords:** hierarchical cluster analysis, principal component analysis, ultrasound-assisted extraction, triterpenic acid, *Ziziphus jujuba*

## Abstract

Triterpenoid compounds are one of the main functional components in jujube fruit. In this study, the optimal process for ultrasound-assisted extraction (UAE) of total triterpenoids from jujube fruit was determined using response surface methodology (RSM). The optimal conditions were as follows: temperature of 55.14 °C, ethanol concentration of 86.57%, time of 34.41 min, and liquid-to-solid ratio of 39.33 mL/g. The triterpenoid yield was 19.21 ± 0.25 mg/g under optimal conditions. The triterpenoid profiles and antioxidant activity were further analyzed. Betulinic acid, alphitolic acid, maslinic acid, oleanolic acid, and ursolic acid were the dominant triterpenoid acids in jujube fruits. Correlation analysis revealed a significant positive correlation between the major triterpenic acids and antioxidant activities. The variations of triterpenoid profiles and antioxidant activity within the jujube fruits and the degree of variation were evaluated by hierarchical cluster analysis (HCA) and principal component analysis (PCA), respectively. The results provide important guidance for the quality evaluation and industrial application of jujube fruit.

## 1. Introduction

Jujube (*Ziziphus jujuba* Mill.), belonging to the Rhamnaceae family, is widespread in Asia, Europe, and America [1]. In China, jujube has been cultivated for 4000 years and there are more than 700 cultivars of the fruits [2]. More than four million tons of jujube fruits are harvested in China per year, which represents 90% of the total yield globally [3]. The fruit has been commonly used in Traditional Chinese Medicine (TMC) for its various pharmacological activities, such as its anticancer, antiepileptic, anti-inflammatory, anti-insomnia, and neuroprotective effects [4,5]. In general, the beneficial effects of health are derived from a variety of bioactive compounds, such as triterpenes, alkaloids, flavonoids, and polysaccharides [6]. 

Triterpenes, belonging to the Phytosterol family, are naturally occurring bioactive components that are commonly found in cereals and vegetables [7]. Modern studies have shown that triterpenes and triterpenic acids, derivatives of pentacyclic triterpenes, have a variety of biological effects, such as antioxidative, anti-inflammatory, anticancer, hepatoprotective, and anti-microbial activities, combined with low toxicity [8,9,10]. Triterpenic acids in jujube fruit have been demonstrated to be a group of major bioactive compounds [11,12,13]. For example, triterpenic acids have been reported to be the most active part in jujuba for the inhibitory effects on inflammatory cells [14]. Alphitolic acid and 3-O-trans-coumaroyl alphitolic acid in jujube can significantly reduce nitric oxide (NO) release and the inducible nitric oxide synthase (iNOS) expression in macrophages [15]. Furthermore, betulinic acid isolated from jujube can cause apoptosis of human breast cancer cell line MCF-7 cells through the mitochondria transduction pathway [16].

The discovery of new natural and safe health products in the form of plant extracts represents a real challenge today. Thus, efficient extraction and further utilization of bioactive triterpenes of jujube and its products have been attracting attention in recent years. [17,18]. To obtain the highest recovery of triterpenoids, it is vital to select the best extraction method and optimize the parameters [19,20]. Compared to conventional extraction methods, such as maceration and Soxhlet extraction, ultrasonic-assisted extraction (UAE) is a green and efficient technology used for its short extraction time, reduced consumption of solvents and energy, and higher extraction yield of bioactive compounds [19,20,21]. This technique has been successfully used to extract triterpene acids from olive pomace [20], pomegranate flowers [22], and *Rosmarinus officinalis* leaves [12]. However, to the best of our knowledge, the application of UAE processes for extracting triterpene compounds from jujube has not been reported before.

Previous research has proved that many factors, such as solvent concentration, extraction temperature, time, and liquid/solid ratio, can affect the extraction efficiency from plant materials. Considering all of these factors and their levels, it is a tedious task to optimize extraction conditions, during which not only does the number of experimental run increase but also the interactive effect cannot be determined [23]. Response surface methodology (RSM) is a statistical method that uses multifactorial modeling to optimize complex processes. It gives a free space wherein the experimental terms can be defined based on the response value, and the levels of factors can be adjusted according to the requirement of the experiment [23,24]. Therefore, this method may be an ideal strategy for the optimization of triterpenoid extraction from jujube.

In addition, the differences in contents of the triterpenes in the materials also affects the composition of the extracts. The compositional profile of bioactive compounds presented in jujube has been found to be influenced by factors, such as cultivar, geographical environment, processing conditions, and storage conditions [23,24,25]. However, because of the difference between the chemical compositions of different cultivars, there are some difficulties in the breeding and planting of jujube varieties, as well as in the quality evaluation and standardization of the developed products. Therefore, it is of great significance for customers and the industry to explore the profiles of triterpenic acids of different jujube cultivars without regional disparity.

The aims of this study were: (1) to optimize the UAE conditions for triterpenoids from jujube fruit using RSM. The effects of extraction temperature, ethanol concentration, time, and the solvent-to-solid ratio on the total triterpenoid yield were studied. (2) To analyze the antioxidant activities and major triterpenic acids profiles in the extracts of different jujube samples. (3) To study the differences in the contents of triterpenic acids and antioxidant activities among different cultivars using principal component analysis (PCA) and hierarchical cluster analysis (HCA). This study provides a comprehensive triterpenoid acid profile of different jujube cultivars, irrespective of the origin differences, and the results provide substantial information on the understanding and utilization of the phytochemical properties of these jujube cultivars for further research.

## 2. Results and Discussion

### 2.1. UAE Process Optimization

#### 2.1.1. Model Fitting

The merits of RSM include the use of a lower number of experimental measurements, the provision of a statistical interpretation of the data, and also the identification of the interaction amongst variables [23,24]. In this study, the Box–Behnken design (BBD) was employed to determine the interactions among *X*_1_ (temperature), *X*_2_ (ethanol concentration), *X*_3_ (time), and *X*_4_ (liquid-to-solid ratio), as well as to optimize the UAE conditions. Table 1 shows the experimental results, and Table 2 summarizes the results of the analysis of variance (ANOVA).

By using multiple regression analysis, the correlation between the response and the tested independent variables was established by Equation (1), and the X_i_ demonstrates the coded variables in the formula.

*Y* = 19.14 + 0.86 *X*_1_ + 0.054 *X*_2_ − 0.020 *X*_3_ + 0.94 *X*_4_ − 0.083 *X*_1_*X*_2_ − 0.13 *X*_1_*X*_3_ − 0.16 *X*_1_*X*_4_ − 0.075 *X*_2_*X*_3_ + 0.065 *X*_2_*X*_4_ − 1.81 *X*_1_^2^ − 0.12 *X*_2_^2^ − 0.31 *X*_3_^2^ − 1.07 *X*_4_^2^ (1)

#### 2.1.2. Model Validation

**Figure 1:**
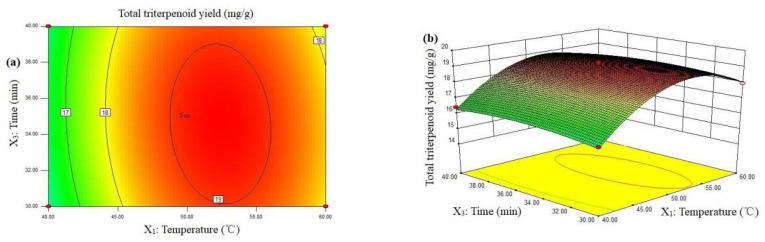

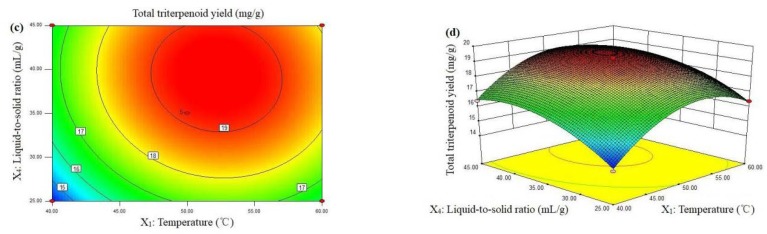
Contour plot (**a**,**c**) and three-dimensional (3D) surface plot (**b**,**d**) showing the interaction effects of the process variables on the total triterpenoid yield. (**a**,**b**): the interaction between temperature (*X*_1_) and time (*X*_3_) on total triterpenoid yield (*Y*); (**c**,**d**): the interaction between temperature (*X*_1_) and liquid-to-solid ratio (*X*_4_) on total triterpenoid yield (*Y*).

The contour plot and the three-dimensional (3D) surface plot response surfaces described by the regression model are represented in Figure 1, and the maximum yield total triterpenoid was recorded under follow conditions: temperature of 55.14 °C, ethanol concentration of 86.57%, time of 34.41 min, and liquid-to-solid ratio of 39.33 mL/g. In order to check if the model was valid, extraction was carried out in triplicate under the optimal conditions. Further, the measured values (19.21 ± 0.25 mg/g) were in the range of the 95% confidence interval (95% CI) of the predicted value (19.44 mg/g), which verifies the predictability of the proposed model.

### 2.2. Triterpenic Acid Contents in the 99 Jujube Samples 

**Figure 2:**
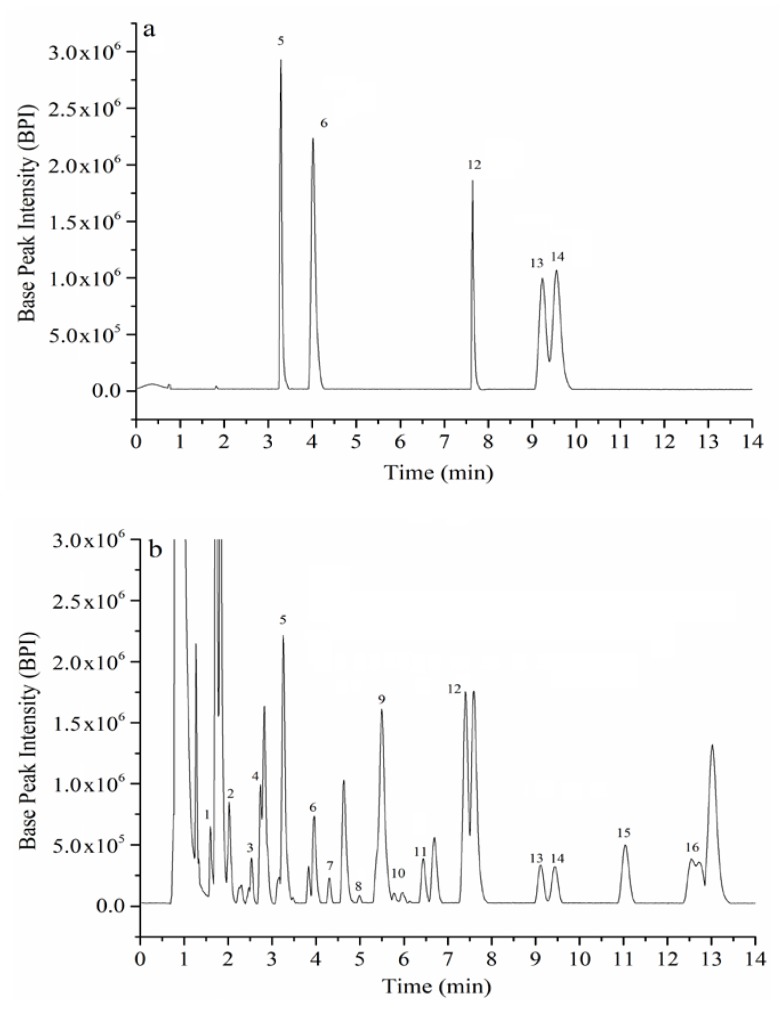
Ultra-performance liquid chromatography (UPLC) chromatograms of mixed standards (**a**) and sample (**b**). 1: Maslinic acid isomer-1 (Ma1); 2: Maslinic acid isomer-2 (Ma2); 3: Maslinic acid isomer-3 (Ma3); 4: Maslinic acid isomer-4 (Ma4); 5: Alphitolic acid (Aa); 6: Maslinic acid (Ma); 7: 2α-hydroxy ursolic acid (2αHa); 8: Maslinic acid isomer-5 (Ma5); 9: Oleanolic acid isomer-1 (Oa1); 10: Maslinic acid isomer-6 (Ma6); 11: Maslinic acid isomer-7 (Ma7); 12: Betulinic acid (Ba); 13: Oleanolic acid (Oa); 14: Ursolic acid (Ua); 15: Betulonic acid (Ba’); 16: Oleanonic acid + Ursonic acid (Oa’ + Ua’).

The triterpenic acids extracted at optimal conditions from 99 cultivars of jujube samples were analyzed by ultra-performance liquid chromatography-mass spectrometry (UPLC–MS). The typical chromatograms of the 99 jujube samples are shown in Figure 2.

**Figure 3:**
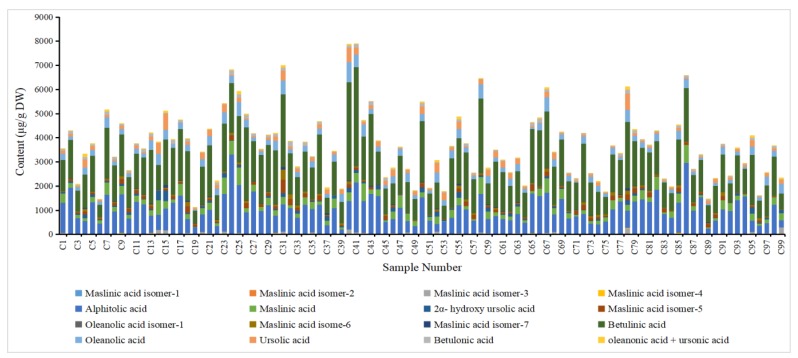
Contents (µg/g dry weight (DW)) of triterpenic acids in different jujube samples.

Sixteen peaks were observed on the chromatogram, including alphitolic acid, maslinic acid, 2α-hydroxy ursolic acid, betulinic acid, oleanolic acid, ursolic acid, betulonic acid, oleanonic acid, and ursonic acid, which were then identified and quantified. The other peaks were preliminarily identified as maslinic acid isomers and one ursolic acid isomer (Table 3). The quantitative results are shown in Figure 3 (detailed data are shown in Table S2). 

The triterpenes, secondary metabolites of plants, are distributed in several peels, leaves, stems and barks of plants, such as birch bark, olive leaves, mistletoe sprouts, clove flower, apple pomace, *Camellia sinensis*, etc. [26]. However, jujube is one of the few fruits with a high content of triterpenes. In this study, a significant difference in the total triterpenic acid content was observed among the jujube cultivars. The total triterpenic acid content ranged from 1082.775 to 7915.451 µg/g dry weight (DW), with a mean value of 3730.970 µg/g DW. Meanwhile, cultivar Jing39 (C41) had the highest content and Wanshuyuanling (C9) the lowest content of triterpenic acid. These results are consistent with previous results (166–6126 µg/g DW) [15].

Until now, more than 15 triterpenoid acids were found in the fruit of jujube [18]. A previous study reported that the identified triterpenic acids, including alphitolic acid, ceanothic acid, maslinic acid, 2a-hydroxyursolic acid, betulinic acid, ursolic acid, betulonic acid, oleanonic acid, and ursonic acid, showed large variations at different stages of growth [15]. In the present study, betulinic acid (516.409–4097.962 µg/g DW), alphitolic acid (198.195–3282.203 µg/g DW), maslinic acid (13.905–751.855 µg/g DW), oleanolic acid (36.696–837.463 µg/g DW), and ursolic acid (5.267–685.325 µg/g DW) were the dominant triterpenoid acids in jujube. Other triterpenoid acids, such as betulonic acid (9.417–304.731 µg/g DW), 2α-hydroxy ursolic acid (0.005–438.165 µg/g DW), and oleanonic acid + ursonic acid (9.834–244.797 µg/g DW), were relatively low. Notably, preliminary findings revealed seven isomers of maslinic acid; however, the detailed structure and chemical formula need to be investigated.

Some by-products of plant raw materials are good sources of triterpenoid acids. For example, olive pomace, a valuable by-product, contains maslinic acid and oleanolic acid in the proportions of 381.200 mg/g and 29.800 mg/g, respectively [20]. The outer bark of birch contains 11.600 mg/g of triterpenoid acids (betulinic + oleanolic) [27]. The extraction of ursolic, oleanolic, and rosmarinic acids in rosemary leaves reached a maximum of 15.800, 12.200, and 15.400 mg/g, respectively [12]. The ursolic acid content in other plant raw materials are as follows: *Calendula officinalis* flowers (20.530 mg/g DW), *Lamii albi flos* (110.400 mg/g DW), *Malus domestica* fruit peel (14.300 mg/g DW), and *Silphium* sp. flowers (17.950–22.050 mg/g DW) [19].

**Figure 4:**
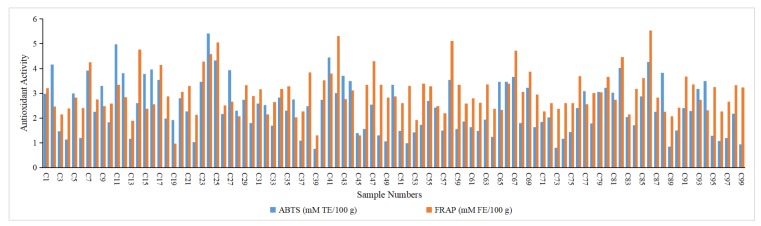
The antioxidant activities of the extracts of different jujube samples. ABTS = 2,2-azinobis (3-ethylbenzothiazoline-6-sulphonic acid) FRAP = ferric reducing antioxidant power kit.

The 2,2-azinobis (3-ethylbenzothiazoline-6-sulphonic acid) (ABTS) and ferric reducing antioxidant power kit (FRAP) assays were carried out to differentiate the antioxidant properties of extracts from different jujube (Figure 4). The ABTS assay is based on hydrogen-donating antioxidants against nitrogen radicals, while the FRAP assay reflects the ferric ion-reducing antioxidant power of antioxidant. These methods have been widely used to evaluate the antioxidant capacity of food extracts [28]. In the present study, the ABTS and FRAP values ranged from 0.753 to 5.421 mM TE/100 g (C24, *Junzao*) and 0.968 to 5.529 mM FE/100 g (C86, *Huizaobianzhongyihao* ), respectively. 

In order to explore the effect of the antioxidant capacity in jujube, correlations among the triterpenic acids and the antioxidant activities were also analyzed (Table 4). As documented in Table 4, a significant positive correlation was observed between ABTS^+^ radical scavenging activity and the contents of alphitolic acid, maslinic acid, betulinic acid, ursolic acid, betulonic acid, and total triterpenic acids (*p* < 0.05). Meanwhile, alphitolic acid, maslinic acid, betulinic acid, oleanolic acid, ursolic acid, betulonic acid, and total triterpenic acids also showed a positive correlation with the FRAP value (*p* < 0.05). 

Obviously, the major triterpenic acids that widely exist in jujube are one of the main antioxidants with various important physiological and pharmacological properties. Previous researchers have proved that pentacyclic triterpenes, such as maslinic acid, alphitolic acid, maslinic acid, oleanolic acid, ursolic acid, glycyrrhetinic acid, betulinic acid, and lupeol, contribute various important physiological and pharmacological properties [29]. For example, ursolic acid and its isomer, oleanolic acid, have been reported have many beneficial effects, such as antioxidative, antimicrobial, anti-inflammatory, anticancer, anti-hyperlipidemic, analgesic, hepatoprotectory, gastroprotective, anti-ulcer, anti-HIV, cardiovascular, antiatherosclerotic, and immunomodulatory effects [19]. Betulinic acid has been reported to have anti-inflammatory, anti-cancer, anti-leukemia, anti-viral, and antihelmintic activities [30]. Due to its selective cytotoxicity against tumor cells and favorable therapeutic index, betulinic acid is considered a promising chemotherapeutic agent against HIV infection and cancers [31]. Maslinic acid has been shown to have antioxidant, anti-inflammatory, antimalarial, and antiprotozoal activities [29]. Therefore, the results of this study provide important guidance for health product development based on jujube.

### 2.3. HCA and PCA

HCA and PCA are effective tools for multivariate analysis, which can be used to explore the existing differences among groups. HCA indicates the similarity among different cultivars, while PCA indicates the significant differences among the cultivars, thus reducing the dimensionality and increasing the interpretability of large datasets [24]. In this study, HCA and PCA were carried out based on the triterpenic acid content and antioxidant activities. 

**Figure 5:**
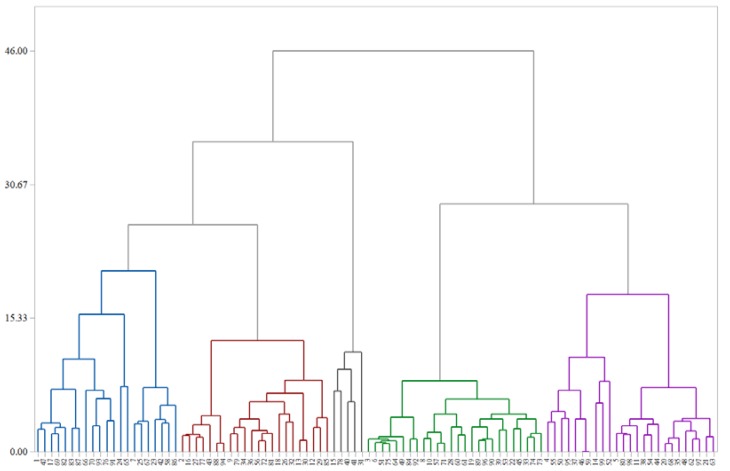
Hierarchical cluster analysis (HCA) of 99 cultivars of jujube samples. Cultivar lines with the same color are in the same cluster.

As shown in Figure 5, the 99 cultivars were divided into five clusters. The mean values of the detected compounds and antioxidant activities in each cluster are listed in Table 5.

Cluster 1 contained 21 samples, which were generally clustered together according to the higher values of alphitolic acid (mean of 1535.713 μg/g DW), maslinic acid (mean of 449.873 μg/g DW), FRAP (mean of 3.931 mM TE/100 g), and lower content of ursolic acid (mean of 92.502 μg/g DW). Cluster 2 consisted of 22 samples, which were the cultivars with the highest content of betulonic acid (mean of 109.488 μg/g DW) and relatively low levels of ursolic acid (mean of 92.296 μg/g DW), respectively. It is noteworthy that cluster 3 consisted of five samples (C15, C31, C40, C41, and C78), in which the mean contents of 2α-hydroxy, betulinic acid, oleanolic acid, ursolic acid, and total triterpenic acids were the highest among those five clusters. The mean values were 216.845, 3158.536, 568.121, 530.525, and 6814.528 μg/g DW, respectively. Accordingly, these samples also had relatively high level of ABTS and FRAP, with mean values of 3.066 and 3.172 mM TE/100 g, respectively. On the contrary, cluster 3 contained 26 samples, which had lower concentrations of most of the major triterpenic acids. For example, the mean levels of alphitolic acid, maslinic acid, 2α-hydroxy ursolic acid, betulinic acid, oleanolic acid, and total triterpenic acids, as well as the antioxidant activities (ABTS and FRAP assay), were the lowest in the five clusters. Meanwhile, the 25 samples in cluster 5 also had relatively lower contents of most compounds.

According to the above results, all of the studied variables might contribute to sample classification. Typically, clusters 3 represented the groups with higher contents of triterpenic acids and higher antioxidant activity, while cluster 4 was indicative of the groups with lower levels.

PCA was carried out to analyze the differences among the 99 cultivars of jujube. The extraction sums of squared loadings are listed in Table 6.

The ellipses of the constant distance of the PCA method were calculated with a 95% confidence interval. Four principal components (PCs) were extracted, and the accumulative contribution rate of the four principal components was 73.53%. PC1, PC2, and PC3 explained 31.79%, 20.46%, and 14.23% of the total variance, together accounting for 66.49% of the total variance (Table 6). From the component matrix of the four principal components (Table S3), it can be inferred that the weight occupied by different triterpenic acids showed significant differences among the different main components. Overall, almost all of the compounds may contribute to the classification of the samples. A reduction in date dimension was successfully achieved. These four principal components, to a large extent, are indicative of the original 18 variables.

In addition, the scatter plot produced by PCA is very important and powerful, since it displays all samples in two- or three-dimensional graphs, and comparisons can be carried out among samples on the basis of the response variables applied in the study [28].

**Figure 6:**
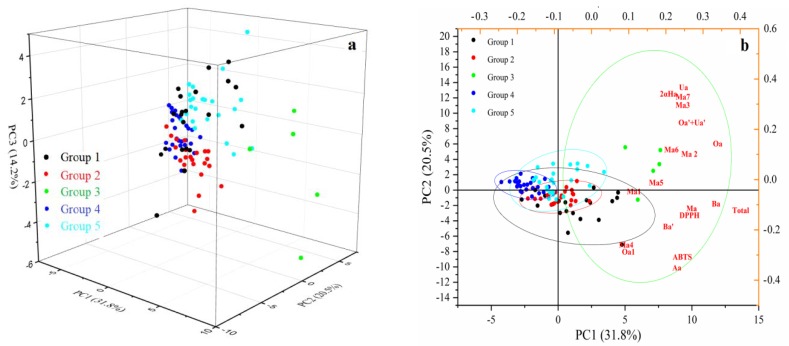
The 3D plots (**a**) and biplots (**b**) of PCA. Groups 1, 2, 3, 4, and 5 are the classified clusters of jujube by HCA. The ellipses with different colors represent the 95% confidence ellipse for different clusters.

The interrelationship between different cultivars is clearly shown in the 3D score plot of PCA (Figure 6a). A significance of differences between groups can be observed in Figure 6b, which shows the profiles of the 95% confidence ellipse for different groups. If there is no intersection between two ellipses, it means that those two groups have significant differences [24]. In this case, since groups 1, 2, and 5 had an intersection between them, they could not be completely distinguished from each other. In other words, because all of the samples were collected from the same origin with similar cultivation conditions, the variability between the samples was not sufficient enough to classify all of the groups accurately. However, groups 3 and 4 were completely separated from each other, which indicates that there is a significant difference between these two groups. This result is consistent with HCA, which revealed that groups 3 and 4 were the cultivars with highest and lowest triterpenic acids contents, respectively. 

Similar results have also been reported by previous researchers when PCA was used to distinguish between different clusters of jujube [24], jujube leaves [32], and finger millets [28]. The results provide important support for cultivation and breeding, quality evaluation, and product development of jujube. Furthermore, research on the main mechanism behind these differences of the different cultivars is urgently needed by molecular biological techniques, such as genomics and enzymology. 

## 3. Materials and Methods 

### 3.1. Plant Materials

Jujube of 99 cultivars (red maturity stage) (Table S1) were picked from the Germplasm Resources Base of Tarim University at Alaer City of Xinjiang Province, China, by the end of October 2019. Fruits without disease and mechanical injury and uniformly shaped were collected randomly from each side of the trees. After harvesting, all samples were lyophilized and then ground to fine powders and stored below –18 °C before analysis. 

### 3.2. Chemicals

The following standards were purchased from ANPEL Co., Ltd (Shanghai, China): alphitolic acid (SPR01052), maslinic acid (Lot 67050010), betulinic acid (B330270), oleanolic acid (Lot Y4430050), and ursolic acid (Lot 40920050). Chemicals, such as acetonitrile, methanol, and ammonium formate, were all of HPLC grade and were purchased from Merck (Darmstadt, Germany). A 2,2-azinobis (3-ethylbenzothiazoline-6-sulphonic acid) (ABTS) kit (Art. No. A015-2) and a ferric reducing antioxidant power kit (FRAP, Art. No. A015-3) were provided by Jiancheng Biology Engineering Institute (Nanjing, Jiangsu, China). Other reagent solutions were of analytical grade (Solarbio Life Sciences Co., Ltd., Beijing, China).

### 3.3. Determination of Total Triterpenoid Content (TTC) 

The TTC was measured using the vanillin–perchloric acid assay method [5]. The results were expressed as oleanolic acid equivalents (OAE, mg/g DW) through the standard calibration curve (*y* = 16.005*x* − 0.0256, *R*^2^ = 0.9987). The total triterpene yield was measured using the following equation:

(2) $$\mathrm{Total}\;\mathrm{triterpene}\;\mathrm{yield}\;\left(\mathrm{mg}/g\right)=\frac{\mathrm{the}\;\mathrm{mass}\;\mathrm{of}\;\mathrm{extracted}\;\mathrm{triterpenes}\left(\mathrm{mg}\right)}{\mathrm{the}\;\mathrm{mass}\;\mathrm{of}\;\mathrm{dried}\;\mathrm{sample}\;\left(g\right)}.$$

### 3.4. Analysis of Triterpenic Acids by UPLC–MS

The extracts (extracted at optimum conditions) from the 99 cultivars of jujube were analyzed using a Waters ACQUITY UPLC H-CLASS system coupled with a Waters Xevo G2-XS QTof (Waters, Milford, MA) [15]. A Waters BEH C18 column (100 × 2.1 mm, 1.7 µm) operated at 30 °C was used. The injection volume was 2.0 µL, and the flow rate was 0.3 mL/min. The mobile phase was composed of A (3 mmol of ammonium formate) and B (methanol mixed with equal volume of acetonitrile) with a gradient elution of 0–2 min, 24–23% A; 2–18 min, 23% A. The parameters for the MS were set as follows: capillary voltage of 3.0 kV, source temperature of 110 °C, desolvation temperature of 450 °C, cone gas flow rate of 50 L/h, and desolvation gas flow rate of 800 L/h. Mass spectra in negative ion modes were recorded within the range of 100–1000 *m/z*. Concentrations of the compounds were calculated using the peak areas of the sample and the corresponding standards.

### 3.5. Analysis of Antioxidant Activities

The antioxidant activities were analyzed according to the methods reported by our lab [33]. Briefly, the samples were extracted according to the optimized extraction process. After centrifugation and lyophilization, the extract was then diluted to 10 mL for further antioxidant activity analysis. The antioxidant activities were evaluated using an ABTS kit and a FRAP kit, and the operational steps were performed in compliance with the instructions of the kits. The ABTS radical scavenging activity was expressed as millimoles of Trolox equivalent per 100 g (mM TE/100 g) of dry sample. The FRAP results were expressed as millimoles of ferrous sulfate equivalent per 100 g (mM FE/100 g) of dry sample.

### 3.6. UAE Procedures

Two grams (2.0 g) of dried jujube powder (C24, Junzao) was placed in an ultrasonic extractor (XY–2008; Xiyu Instruments Co., Ltd., Shanghai, China) at different influencing factors, including temperature (30–70 °C), ethanol concentration (55–95%), time (20–40 min), and liquid-to-solid ratio (15:1–55:1 mL/min). After extraction, the extracts were centrifuged at the speed of 5000 rpm for 5 min (Centrifuge 5804; Eppendorf AG, Germany). Then, the supernatants were evaporated, lyophilized, and stored below −18 °C until further analysis. Based on the results of single-factor experiments, the factors that have a major influence and the levels of those influences were determined and applied in the RSM design.

### 3.7. RSM Experimental Design

A four-factor three-level experimental RSM was employed to determine the optimal conditions. The yield of total triterpenoids (*y*, mg/g) was regarded as a dependent variable. Furthermore, the four-factor ranges were determined according to the previous single-factor experiments (data not shown). Table 7 shows the experimental design.

### 3.8. Statistical Analysis

Design Expert 8.0.5 (Stat-Ease Inc., Minneapolis, MN, USA) was used to analyze RSM optimization and regression. The values of the dependent parameters obtained from the experiments were fitted to the second-order polynomial model as shown below:

(3) $$Y=\beta_{0}+\sum_{i=1}^{k}\beta_{i}X_{i}+\sum_{i=1}^{k}\beta_{ii}X_{i}^{2}+\sum_{i=1}^{k-1}\sum_{j>1}^{k}\beta_{ij}X_{i}X_{j}.$$

*Y* stands for the estimated response, *X_j_* and *X_i_* represent the independent variables, while *k* suggests variable number. Meanwhile, *β_i_*, *β*_0_, *β_ij_*, and *β_ii_* represent the regression coefficients of the linear, intercept, interaction, and quadratic terms, respectively. 

All data were collected in triplicate and expressed as mean ± SD. The one-way ANOVA analysis, HCA, and PCA were carried out by SPSS 22.0 software (SPSS, Chicago, IL, USA). 

## 4. Conclusions

In this study, the ultrasound-assisted extraction of total triterpenoids from jujube was optimized by RSM. The optimal conditions obtained were as follows: temperature of 55.14 °C, ethanol concentration of 86.57%, time of 34.41 min, and liquid-to-solid ratio of 39.33 mL/g. The triterpenoid yield was 19.21 ± 0.25 mg/g under the optimal conditions. 

The triterpenoid acid profile of the extracts obtained from 99 cultivars of jujube were further analyzed by UPLC–MS. Betulinic acid (mean of 1602.008 μg/g DW), alphitolic acid (mean of 1017.060 μg/g DW), maslinic acid (mean of 265.568 μg/g DW), oleanolic acid (mean of 264.445 μg/g DW), ursolic acid (mean of 151.166 μg/g DW), betulonic acid (mean of 69.570 μg/g DW), and 2α-hydroxy ursolic acid (mean of 66.032 μg/g DW) were found to be the main triterpenoid acids in jujube of different cultivars. According to HCA and PCA, the 99 cultivars were categorized into five clusters, among which cluster 3 had relatively higher contents of most triterpenoid acids. 

These results indicate that jujube is a potential natural source of triterpenic acids for the development of functional foods, and the differences in the compositional profile of cultivars may lead to their different applications. UAE is an efficient method to extract triterpenoids from jujube, and RSM is a useful method to optimize the UAE parameters of triterpenoid compounds from jujube. This study would be further contributable for the deep processing and utilization of jujube.

## Figures and Tables

**Table 1:** The experimental results.

Run	*X*_1_: Temperature (°C)	*X*_2_: Ethanol Concentration (%)	*X*_3_: Time (min)	*X*_4_: Liquid-to-Solid Ratio (mL/g)	*Y*: Total Triterpenoid Yield (mg/g)
1	40	90	35	35	16.60
2	50	80	40	35	18.68
3	40	85	35	25	14.13
4	60	85	40	35	17.72
5	50	85	30	25	16.82
6	40	85	35	45	16.40
7	50	85	35	35	19.25
8	40	85	30	35	16.13
9	50	80	35	25	17.18
10	60	80	35	35	17.96
11	50	85	35	35	19.18
12	50	90	40	35	18.58
13	60	90	35	35	18.08
14	50	90	35	45	18.93
15	50	85	35	35	19.12
16	50	85	40	25	16.78
17	50	80	35	45	18.81
18	50	90	30	35	18.84
19	40	80	35	35	16.15
20	60	85	35	45	18.01
21	50	80	30	35	18.64
22	50	90	35	25	17.04
23	50	85	30	45	18.73
24	50	85	35	35	19.05
25	60	85	30	35	17.95
26	40	85	40	35	16.42
27	50	85	35	35	19.08
28	50	85	40	45	18.69
29	60	85	35	25	16.38

**Table 2:** Analysis of variance (ANOVA) for the response surface quadratic model.

Source	Sum of Squares	df	Mean Square	*F* Value	*p*-value
Model	45.29	14	3.24	229.64	<0.0001
*X*_1_: Temperature	8.79	1	8.79	623.9	<0.0001
*X*_2_: Ethanol concentration	0.035	1	0.035	2.5	0.1362
*X*_3_: Time	0.0048	1	0.0048	0.34	0.5687
*X*_4_: Liquid-to-solid ratio	10.53	1	10.53	747.33	<0.0001
*X*_1_ *X*_2_	0.027	1	0.027	1.93	0.1862
*X*_1_ *X*_3_	0.068	1	0.068	4.8	0.0459
*X*_1_ *X*_4_	0.1	1	0.1	7.27	0.0174
*X*_2_ *X*_3_	0.023	1	0.023	1.6	0.2269
*X*_2_ *X*_4_	0.017	1	0.017	1.2	0.2919
*X*_3_ *X*_4_	0	1	0	0	1
*X*_1_^2^	21.3	1	21.3	1512.04	<0.0001
*X*_2_^2^	0.089	1	0.089	6.32	0.0248
*X*_3_^2^	0.61	1	0.61	43.09	<0.0001
*X*_4_^2^	7.37	1	7.37	523.14	<0.0001
Residual	0.2	14	0.014		
Lack of Fit	0.17	10	0.017	2.67	0.1785
Pure Error	0.026	4	0.00643		
Cor Total	45.49	28			
Adeq Precision	56.589				
R^2^ = 0.9957; Adj R^2^ = 0.9913; Pred R^2^ = 0.9774					

ANOVA can fully reflect the significance and reliability of the response surface quadratic regression model [23,24]; as indicated in Table 2, the model was highly significant (*F* = 229.64, *p* < 0.0001). The *p*-value for the lack of fit was not significant (*F* = 2.67, *p* = 0.1785), which indicates the adequate predictive relevance of the model to explain the associations of independent variables with dependent variables. The linear coefficients (*X*_1_, *X*_4_), quadratic coefficients (*X*_1_^2^, *X*_2_^2^, *X*_3_^2^, and *X*_4_^2^), and interaction coefficients (*X*_1_ *X*_3_, *X*_1_ *X*_4_) were significant (*p* < 0.05). The R^2^ value of 0.9957 indicates a reasonable fit of the model to the experimental data. An *R*^2^ value (multiple correlation coefficient) closer to one denotes better correlation between the observed and predicted values. In this study, the values of *R*^2^ (0.9957), Pred *R*^2^ (0.9774), and Adj *R*^2^ (0.9913) indicate a good correlation between the experimental and predicted values, which shows that the model was significant. In addition, “Adeq Precision” (a measure of the signal-to-noise ratio) of 56.589 indicates an adequate signal. It can be concluded that the model was statistically credible and reliable.

**Table 3:** The retention time, mass spectrum (MS) parameters, and regression equations of standards and isomers.

Peak No.	Retention Time (min)	Compound	[M + H]^−^ (*m/z*)	Regression Equation	R^2^
1	1.72	Maslinic acid isomer-1	471.34	—	—
2	2.16	Maslinic acid isomer-2	471.34	—	—
3	2.56	Maslinic acid isomer-3	471.34	—	—
4	2.76	Maslinic acid isomer-4	471.34	—	—
5	3.29	Alphitolic acid	471.34	y = 138799x + 1595.7	0.9994
6	3.99	Maslinic acid	471.34	y = 140553x + 1065.1	0.9996
7	4.34	2α-hydroxy ursolic acid	471.34	—	—
8	5.04	Maslinic acid isomer-5	471.34	—	—
9	5.41	Oleanolic acid isomer-1	455.35	—	—
10	5.72	Maslinic acid isomer-6	471.34	—	—
11	6.3	Maslinic acid isomer-7	471.34	—	—
12	7.64	Betulinic acid	455.35	y = 125572x + 1121.5	0.9991
13	9.17	Oleanolic acid	455.35	y = 90033x - 1164.7	0.9992
14	9.49	Ursolic acid	455.35	y = 113372x + 1035.8	0.9997
15	11.09	Betulonic acid	455.35	—	—
16	12.59	Oleanonic acid + Ursonic acid	455.35	—	—

**Table 4:** Correlation coefficients (r) of the studied triterpenic acids and the antioxidant activity of jujube cultivars.

	Ma1	Ma 2	Ma3	Ma4	Aa	Ma	2αHa	Ma5	Oa1	Ma6	Ma7	Ba	Oa	Ua	Ba’	Oa’ + Ua’	Total	ABTS	DPPH
ABTS	0.1274	0.1943	–0.0398	0.2576 ^a^	0.9285 ^b^	0.5205 ^b^	0.0146	0.1462	–0.0792	–0.0141	–0.051	0.4838 ^b^	0.1736	0.3378 ^b^	0.464 ^b^	–0.009	0.694 ^b^	-	0.5205 ^b^
FRAP	0.1174	0.0713	0.2629	0.1938	0.5549 ^b^	0.9475 ^b^	0.1955	–0.1852	0.0911	–0.013	–0.0387	0.3508 ^b^	0.6123 ^b^	0.2048 ^a^	0.0111	0.1888	0.583 ^b^	0.4993 ^b^	-

^a^ Significant at *p* < 0.05. ^b^ Significant at *p* < 0.01.

**Table 5:** The mean values of the detected compounds in different clusters.

Variables	Cluster 1	Cluster 2	Cluster 3	Cluster 4	Cluster 5
Maslinic acid isomer-1	**0.046 ^b^**	0.022	0.040	**0.011 ^a^**	0.020
Maslinic acid isomer-2	0.355	1.706	**7.917 ^b^**	0.332	**0.266 ^a^**
Maslinic acid isomer-3	25.352	17.883	**161.294 ^b^**	**6.081 ^a^**	65.097
Maslinic acid isomer-4	**11.305 ^b^**	2.839	2.802	0.655	**0.316 ^a^**
Alphitolic acid	**1535.713 ^b^**	1245.100	1189.970	**555.637 ^a^**	826.015
Maslinic acid	**449.873 ^b^**	203.267	328.307	**136.641 ^a^**	287.112
2α-hydroxy ursolic acid	40.014	29.682	**216.845 ^b^**	**29.278 ^a^**	127.938
Maslinic acid isomer-5	82.301	149.580	**216.391 ^b^**	79.385	**49.260 ^a^**
Oleanolic acid isomer-1	**12.814 ^b^**	8.929	7.839	**7.487 ^a^**	8.074
Maslinic acid isomer-6	49.880	74.380	**160.215 ^b^**	51.110	38.100 ^a^
Maslinic acid isomer-7	**2.833 ^a^**	9.938	**70.101 ^b^**	3.801	16.411
Betulinic acid	1827.349	1909.479	**3158.536 ^b^**	**1043.670 ^a^**	1411.514
Oleanolic acid	317.869	217.850	**568.121 ^b^**	**159.657 ^a^**	308.816
Ursolic acid	**92.502 ^a^**	**92.296 ^a^**	**530.525 ^b^**	112.549	216.542
Betulonic acid	78.186	**109.488 ^b^**	107.236	48.038	**42.063 ^a^**
Oleanonic acid + Ursonic acid	40.246	33.499	**86.389 ^b^**	**26.385 ^a^**	50.759
Total	4572.323	4104.121	**6814.528 ^b^**	**2258.410 ^a^**	3448.962
ABTS	3.346	2.938	**3.066 ^b^**	**1.488 ^a^**	2.132
FRAP	**3.931 ^b^**	2.645	3.172	**2.336 ^a^**	3.208

Extreme values are in bold; ^a^ the element with the lowest mean value among the five clusters; ^b^ the highest mean value.

**Table 6:** Total variance explained by principal component analysis (PCA).

Component	Eigenvalue	Percentage of Variance (%)	Cumulative (%)
PC1	6.04	31.79	31.79
PC2	3.89	20.46	52.25
PC3	2.70	14.23	66.49
PC4	1.34	7.04	73.53

**Table 7:** Independent variable codes and levels in experimental design.

Code	*X*_1_: Temperature (°C)	*X*_2_: Ethanol Concentration (%)	*X*_3_: Time (min)	*X*_4_: Liquid-to-solid Ratio (mL/g)
−1	40	80	30	25:1
0	50	85	35	35:1
+1	60	90	40	45:1

## Supplementary Materials

The following are available online at https://www.mdpi.com/2223-7747/9/4/412/s1. Table S1. Summary of the information of jujube samples; Table S2. Contents (μg/g DW) of triterpenic acids in jujube fruit samples; Table S3. Component matrix of principal component analysis.

## References

[1] Wang R., Ding S., Zhao D., Wang Z., Wu J., Hu X. (2016). Effect of dehydration methods on antioxidant activities, phenolic contents, cyclic nucleotides, and volatiles of jujube fruits. Food Sci. Biotechnol..

[2] Wang Y.K., Li D.K., Sui C.L., Zhao A.L., Du X.M. (2008). Conservation, characterization, evaluation and utilization of Chinese jujube germplasm resources. Int. Jujube Symp..

[3] Wang J., Nakano K., Ohashi S., Kubota Y., Takizawa K., Sasaki Y. (2011). Detection of external insect infestations in jujube fruit using hyperspectral reflectance imaging. Biosyst. Eng..

[4] Choi S.H., Ahn J.B., Kim H.J., Im N.K., Kozukue N., Levin C.E., Friedman M. (2012). Changes in free amino acid, protein, and flavonoid content in jujube (*Ziziphus* jujube) fruit during eight stages of growth and antioxidative and cancer cell inhibitory effects by extracts. J. Agric. Food Chem..

[5] Kou X.H., Chen Q., Li X.H., Li M.F., Kan C., Chen B.R., Zhang Y., Xue Z.H. (2015). Quantitative assessment of bioactive compounds and the antioxidant activity of 15 jujube cultivars. Food Chem..

[6] San B., Yildirim A.N. (2010). Phenolic, alpha-tocopherol, beta-carotene and fatty acid composition of four promising jujube (*Ziziphus jujuba* Miller) selections. J. Food Compos. Anal..

[7] Siddique H.R., Saleem M. (2011). Beneficial health effects of lupeol triterpene: A review of preclinical studies. Life Sci..

[8] Fujiwara Y., Hayashida A., Tsurushima K., Nagai R., Yoshitomi M., Daiguji N., Sakashita N., Takeya M., Tsukamoto S., Ikeda T. (2011). Triterpenoids isolated from *Zizyphus* jujuba inhibit foam cell formation in macrophages. J. Agric. Food Chem..

[9] Miao L.J., Liu M.J., Liu X.G., Geng J.N., Wang J., Ning Q. (2008). Study on the extraction of triterpenoids from jujube. J. Agric. Univ. Hebei.

[10] Bernatoniene J., Cizauskaite U., Ivanauskas L., Jakstas V., Kalveniene Z., Kopustinskiene D.M. (2016). Novel approaches to optimize extraction processes of ursolic, oleanolic and rosmarinic acids from *Rosmarinus officinalis* leaves. Ind. Crop. Prod..

[11] Guo S., Duan J.A., Tang Y.P., Yang N.Y., Qian D.W., Su S.L., Shang E.X. (2010). Characterization of triterpenic acids in fruits of *ziziphus* species by HPLC-ELSD-MS. J. Agric. Food Chem..

[12] Junhai L., Hongguang G.E., Zhizhou L., Feng N. (2012). Methods for Extracting Ursolic Acid from Red Jujube. China Patent.

[13] Guo S., Duan J.A., Qian D.W., Tang Y.P., Wu D.W., Su S.L., Wang H.Q., Zhao Y.N. (2015). Content variations of triterpenic acid, nucleoside, nucleobase, and sugar in jujube (*Ziziphus* jujuba) fruit during ripening. Food Chem..

[14] Gao Q.H., Wu C.S., Wang M. (2013). The jujube (*Ziziphus jujuba* mill.) fruit: A review of current knowledge of fruit composition and health benefits. J. Agric. Food Chem..

[15] Masullo M., Montoro P., Autore G., Marzocco S., Pizza C., Piacente S. (2015). Quali-quantitative determination of triterpenic acids of *Ziziphus* jujuba fruits and evaluation of their capability to interfere in macrophages activation inhibiting NO release and iNOS expression. Food Res. Int..

[16] Sun Y.F., Song C.K., Viernstein H., Unger F., Liang Z.S. (2013). Apoptosis of human breast cancer cells induced by microencapsulated betulinic acid from sour jujube fruits through the mitochondria transduction pathway. Food Chem..

[17] Cao Y.P., Yang X.L., Xue C.H. (2007). Study on extraction technology of oleanolic acid in *Zizyphus* jujuba date. Food Sci..

[18] Zhang H.Q., Liu P., Duan J.A., Dong L., Shang E.X., Qian D.W., Xiao P., Zhao M., Li W.W. (2019). Hierarchical extraction and simultaneous determination of flavones and triterpenes in different parts of *Trichosanthes kirilowii Maxim*. By ultra-high-performance liquid chromatography coupled with tandem mass spectrometry. J. Pharm. Biomed..

[19] López-Hortas L., Pérez-Larrán P., González-Muñoz M.J., Falqué E., Domínguez H. (2018). Recent developments on the extraction and application of ursolic acid. A review. Food Res. Int..

[20] Xie P.J., Huang L.X., Zhang C.H., Deng Y.J., Wang X.J., Cheng J. (2019). Enhanced extraction of hydroxytyrosol, maslinic acid and oleanolic acid from olive pomace: Process parameters, kinetics and thermodynamics, and greenness assessment. Food Chem..

[21] Wen C.T., Zhang J.X., Zhang H.H., Dzah C.S., Zandile M., Duan Y.Q., Ma H.L., Luo X.P. (2018). Advances in ultrasound assisted extraction of bioactive compounds from cash crops—A review. Ultrason. Sonochem..

[22] Fu Q., Zhang L., Cheng N., Jia M., Zhang Y. (2014). Extraction optimization of oleanolic and ursolic acids from pomegranate (*Punica granatum* L.) flowers. Food Bioprod. Process..

[23] Li J.W., Fan L.P., Ding S.D., Ding X.L. (2007). Nutritional composition of five cultivars of chinese jujube. Food Chem..

[24] Wang L.N., Fu H.Y., Wang W.Z., Wang Y.Q., Zheng F.P., Ni H., Chen F. (2018). Analysis of reducing sugars, organic acids and minerals in 15 cultivars of jujube (*Ziziphus jujuba* mill.) fruits in China. J. Food Compos. Anal..

[25] Gao Q.H., Wu P.T., Liu J.R., Wu C.S., Parry J.W., Wang M. (2011). Physicochemical properties and antioxidant capacity of different jujube (*Ziziphus jujuba* Mill.) cultivars grown in loess plateau of China. Sci. Hortic..

[26] Jäger S., Trojan H., Kopp T., Laszczyk M., Scheffler A. (2009). Pentacyclic triterpene distribution in various plants-rich sources for a new group of multi-potent plant extracts. Molecules.

[27] Popov S.A., Sheremet O.P., Kornaukhova L.M., Grazhdannikov A.E., Shults E.E. (2017). An approach to effective green extraction of triterpenoids from outer birch bark using ethyl acetate with extractant recycle. Ind. Crop. Prod..

[28] Xiang J.L., Li W.H., Ndolo V.U., Beta T. (2019). A comparative study of the phenolic compounds and in vitro antioxidant capacity of finger millets from different growing regions in *Malawi*. J. Cereal Sci..

[29] Sharma H., Kumar P., Deshmukh R.R., Bishayee A., Kumar S. (2018). Pentacyclic triterpenes: New tools to fight metabolic syndrome. Phytomedicine.

[30] Chen Q.H., Liu J., Zhang H.F., He G.Q., Fu M.L. (2009). The betulinic acid production from betulin through biotransformation by fungi. Enzyme Microb. Technol..

[31] Cichewicz R.H., Kouzi S.A. (2004). Chemistry, biological activity, and chemotherapeutic potential of betulinic acid for the prevention and treatment of cancer and HIV infection. Med. Res. Rev..

[32] Song L.J., Zheng J., Zhang L., Yan S.J., Huang W.J., He J., Liu P.Z. (2019). Phytochemical profiling and eingerprint analysis of Chinese iujube (*Ziziphus jujuba* Mill.) leaves of 66 cultivars from Xinjiang province. Molecules.

[33] Song L.J., Liu P.Z., Yan Y.Z., Huang Y., Bai B.Y., Hou X.J., Zhang L. (2019). Supercritical CO_2_ fluid extraction of flavonoid compounds from Xinjiang jujube (*Ziziphus jujuba* Mill.) leaves and associated biological activities and flavonoid compositions. Ind. Crop. Prod..

