# Supplementary material for: Optimized Extraction of Total Triterpenoids from Jujube (Ziziphus jujuba Mill.) and Comprehensive Analysis of Triterpenic Acids in Different Cultivars
Source: Plants (Basel). 2020 Mar 27;9(4):412. doi: 10.3390/plants9040412 (PMC7238538; doi:10.3390/plants9040412)
Supplement: Supplementary file 1 [file plants-09-00412-s001.pdf]

Supplementary material

Optimized extraction of total triterpenoids from jujube (*Ziziphus jujuba* Mill.) and comprehensive analysis of triterpenic acids in different cultivars

Lijun Song [1], Li Zhang [2], Long Xu [1], Yunjian Ma [1], Weishuai Lian [1], Yongguo Liu [3], Yonghua Wang [1] *

[1] School of Food Science and Engineering, South China University of Technology, Guangzhou, Guangdong 510641, PR China; slj176@163.com (L.S.); xulong19891026@163.com (L.X.); 605740779@qq.com (Y.M.); lianws_scut@163.com (W.L.); yonghw@scut.edu.cn (Y.W.).

[2] College of Life Science, Tarim University, Alar, Xinjiang, 843300, PR China; cxbh1984@163.com (L.Z.).

[3] Beijing Advanced Innovation Centre for Food Nutrition and Human Health, Beijing Key Laboratory of Flavor Chemistry, Beijing Technology and Business University, Beijing 100048, China; liuyg@th.btbu.edu.cn (L.G.).

* Correspondence: yonghw@scut.edu.cn (Y.W.)

Table S1 Summary of the information of jujube samples

No.	Cultivars	No.	Cultivars	No.	Cultivars
C1	Linyixiaozao	C34	Hongshiyihao	C67	Dongjinsanhao
C2	Zhongzaoyihao	C35	Lantiandazao	C68	Tengzhoutangzao
C3	Donglingwuhe	C36	Fuxiang	C69	Junyi
C4	Sanlengzao	C37	Longzao	C70	S-182
C5	Jinsimi	C38	Bopizao	C71	Zaohuangchangzao
C6	Goutouzao	C39	Shisishi	C72	Bayuehong
C7	Mayabai	C40	Jinzaoyihao	C73	Lingzao
C8	Hetaowen	C41	Jing39	C74	Jiulingwu
C9	Wanshuyuanling	C42	Shandongpingguoerhao	C75	Dalilinglingzao
C10	Jiuzhuangwozao	C43	Jikangyihao	C76	Henanyouxiyihao
C11	Yuanlingxinyihao	C44	Junzaobianzhong-1	C77	Momoza
C12	Changjixin	C45	Junzaobianzhonmg-2	C78	Xiangfenyuanzao
C13	Naitouzao	C46	Wubaodazao	C79	Linyilajiaozao
C14	Youcuizao	C47	Yongchengchanghong	C80	Xinzhenglingzao
C15	Bayangzao	C48	Lucui	C81	Xinzhenghongsanhao
C16	Zanyuzao	C49	Gutouzao	C82	Mopanzao
C17	Banzao	C50	Hongdayihao	C83	Zaocuiwang

C18	Houtouzao	C51	Jinsixiaozao	C84	Liuyeuxian
C19	Fushuai	C52	Liuyuehong	C85	Shaungren
C20	Gagazao	C53	Shanghaibaipu	C86	Huizaobianzhongyihao
C21	Yashizao	C54	Goutouzao-2	C87	Xinzhenghongyihao
C22	Tailihong	C55	Zhongyangmuzao	C88	Aijinyihao
C23	Guanyinzao	C56	Shiyierwu	C89	Wuhezao
C24	Junzao	C57	Lajiaozao	C90	Dalizhizao
C25	Zhongzaosanhao	C58	Chahuzao	C91	Pinbazao
C26	Ningyanglingzao	C59	Binlangzao	C92	Dabailing
C27	Luzaowuhao	C60	Jinsiyihao	C93	Luzaoqihao
C28	Luzaosanhao	C61	Changzicui	C94	Lingbaodazao
C29	Jing60	C62	Baodeyouzao	C95	Zanjing
C30	Dongjinyihao	C63	Hengyangzhenzhu	C96	Jinsixinsihao
C31	Hongzhaoshiyuehong	C64	Xiangfenmuzao	C97	Popozao
C32	Yongjijidan	C65	Shupujianzao	C98	Luodihong
C33	Shyishiqi	C66	Changjixin	C99	Aiguochanghong

Table S2. Contents (μg/g DW) of triterpenic acids in jujube samples

No.[a]	Content of triterpenic acids (μg/g DW) [b]																
	Maslinic acid isomer-1 [c]	Maslinic acid isomer-2 [c]	Maslinic acid isomer-3 [c]	Maslinic acid isomer-4 [c]	Alphitolic acid	Maslinic acid	2α-hydroxy ursolic acid [c]	Maslinic acid isomer-5 [c]	Oleanolic acid isomer-1[d]	Maslinic acid isome-6 [c]	Maslinic acid isomer-7 [c]	Betulinic acid	Oleanolic acid	Ursolic acid	Betulonic acid [e]	oleanonic acid + ursonic acid [d]	Total
C1	0.013 ± 0	0.026 ± 0.005	61.919 ± 2.801	13.265 ± 1.110	1243.954 ± 72.225	372.041 ± 30.887	52.891 ± 5.228	27.306 ± 2.011	13.904 ± 0.594	21.273 ± 1.771	0.893 ± 0.055	1273.537± 77.329	267.806 ± 21.884	116.674 ± 15.224	72.111 ± 11.119	36.452 ± 1.584	3574.064 ± 243.827
C2	0.027 ± 0.001	0.039 ± 0.001	35.296 ± 1.352	0.019 ± 0	1906.380 ± 91.220	194.417 ± 16.375	61.183 ± 2.014	79.571 ± 10.221	7.401 ± 0.653	21.063 ± 1.235	6.769 ± 0.459	1598.035 ± 82.357	146.65 ± 12.598	94.328 ± 8.359	128.435 ± 11.358	28.377 ± 1.986	4307.989 ± 240.189
C3	0.006 ± 0	0.020 ± 0	0.683 ± 0.002	0.031 ± 0.001	678.929 ± 50.237	139.819 ± 10.205	42.044 ± 3.365	45.812 ± 3.652	7.401 ± 0.438	28.427 ± 2.587	2.401 ± 0.165	867.58 ± 60.824	138.237 ± 10.582	40.073 ± 1.975	55.495 ± 4.658	39.839 ± 2.621	2086.794 ± 151.312
C4	0.009 ± 0	0.036 ± 0.001	32.920 ± 2.312	0.021 ± 0	517.342 ± 46.270	123.775 ± 11.023	154.219 ± 11.249	116.959 ± 8.952	7.734 ± 0.643	100.158 ± 9.326	51.4 ± 4.284	1377.632 ± 75.952	289.97 ± 20.536	322.753 ± 24.327	100.184 ± 7.842	149.46 ± 9.985	3344.57 ± 232.702
C5	0.010 ± 0	0.003 ± 0	12.113 ± 0.103	0.019 ± 0	1317.990 ± 70.356	220.664 ± 18.652	108.88 ± 7.508	67.949 ± 5.349	7.684 ± 0.852	29.156 ± 2.643	1.64 ± 0.095	1487.37 ± 85.687	248.479 ± 19.853	144.044 ± 8.689	71.45 ± 5.692	47.353 ± 3.129	3761.524 ± 228.608
C6	0.006 ± 0	0.035 ± 0.001	37.630 ± 1.450	0.049 ± 0.001	407.846 ± 35.571	132.548 ± 10.250	49.561 ± 3.592	25.524 ± 2.038	8.123 ± 0.670	37.964 ± 2.958	1.394 ± 0.095	523.329 ± 40.386	140.741 ± 10.358	53.625 ± 4.627	24.012 ± 1.956	31.481 ± 2.353	1473.867 ± 116.306
C7	0.027 ± 0	0.055 ± 0.002	11.696 ± 0.484	0.654 ± 0.002	1624.611 ± 90.791	505.247 ± 32.432	54.997 ± 4.925	41.091 ± 3.267	9.034 ± 0.753	49.636 ± 3.651	2.917 ± 0.185	2111.78 ± 123.650	431.107 ± 26.523	112.026 ± 10.352	138.886 ± 9.637	90.043 ± 8.531	5183.807 ± 315.185
C8	0.019 ± 0.001	0.768 ± 0.001	3.774 ± 0.035	0.022 ± 0	947.265 ± 57.068	188.245 ± 9.683	50.021 ± 4.327	111.555 ± 9.278	6.468 ± 0.428	45.965 ± 3.841	10.007 ± 0.867	1494.262 ± 98.362	173.862 ± 10.324	57.093 ± 4.267	108.964 ± 7.395	26.611 ± 1.954	3214.901 ± 207.831
C9	0.032 ± 0	0.885 ± 0.001	55.580 ± 4.582	2.547 ± 0.034	1598.141 ± 83.650	342.180 ± 15.734	100.126 ± 9.624	189.213 ± 20.374	8.778 ± 0.722	100.641 ± 8.330	14.315 ± 0.998	1726.954 ± 100.350	223.094 ± 18.667	134.395 ± 8.352	75.371 ± 5.325	39.478 ± 2.455	4611.73 ± 279.198

C10	0.028 ± 0	0.058 ± 0.001	0.019 ± 0	0.008 ± 0	663.073 ± 40.421	156.307 ± 8.568	101.797 ± 9.378	135.849 ± 10.841	6.468 ± 0.513	60.998 ± 5.220	0.006 ± 0	1249.146 ± 90.314	127.446 ± 8.752	51.062 ± 3.241	73.966 ± 0.327	20.374 ± 1.780	2646.605 ± 179.356
C11	0.020± 0.000	0.500 ± 0.005	44.986 ± 1.357	0.650 ± 0.005	1310.665 ± 85.432	248.806 ± 11.023	90.474 ± 6.374	123.526 ± 8.267	8.64 ± 0.133	65.669 ± 3.240	7.872 ± 0.435	1441.498 ± 100.000	205.412 ± 18.645	138.007 ± 10.527	60.793 ± 4.325	30.398 ± 1.638	3777.916 ± 251.406
C12	0.031 ± 0.001	7.043 ± 0.800	0.014 ± 0	0.016 ± 0	1230.471 ± 65.280	210.294 ± 9.038	20.022 ± 1.052	113.43 ± 9.248	6.912 ± 0.524	57.946 ± 4.287	10.007 ± 0.740	1529.732 ± 101.340	172.246 ± 10.327	56.79 ± 3.278	118.721 ± 7.550	31.22 ± 1.421	3534.898 ± 214.886
C13	0.023 ± 0.001	0.946 ± 0.002	51.325 ± 3.358	7.733 ± 0.597	723.453 ± 15.681	218.600 ± 8.358	50.472 ± 4.217	116.628 ± 7.358	14.526 ± 0.952	67.067 ± 4.225	22.166 ± 1.020	2217.58 ± 198.140	397.386 ± 30.511	165.959 ± 11.250	110.624 ± 8.313	51.807 ± 1.225	4216.296 ± 295.208
C14	0.007 ± 0	0.040 ± 0.002	190.344 ± 7.324	ND	619.848 ± 13.217	612.932 ± 12.138	397.206 ± 15.250	3.457 ± 0.244	6.907 ± 0.421	17.1 ± 1.032	12.354 ± 0.955	1005.768 ± 88.320	477.962 ± 30.142	442.69 ± 30.240	9.417 ± 0.720	19.425 ± 1.203	3815.457 ± 201.208
C15	0.020 ± 0	7.509 ± 0.431	166.072 ± 6.873	0.047 ± 0.001	890.561 ± 18.365	320.231 ± 10.753	438.165 ± 20.523	57.519 ± 3.265	7.901 ± 0.352	41.575 ± 3.521	104.537 ± 8.623	1894.021 ± 162.340	395.281 ± 25.355	685.325 ± 50.452	23.395 ± 1.882	85.684 ± 7.263	5117.841 ± 319.999
C16	0.019 ± 0	0.046 ± 0.002	5.606 ± 0.658	1.729 ± 0.023	1306.183 ± 95.960	147.026 ± 5.651	13.219 ± 0.853	115.173 ± 9.647	9.445 ± 0.842	41.738 ± 2.531	10.045 ± 0.753	1926.475 ± 100.622	162.972 ± 10.350	23.68 ± 1.855	146.184 ± 9.840	35.08 ± 2.752	3934.619 ± 242.339
C17	0.020 ± 0	0.050 ± 0.002	0.015 ± 0.001	19.939 ± 0.681	1592.459 ± 87.657	482.818 ± 18.354	20.02 ± 1.420	39.814 ± 2.482	11.5 ± 0.741	46.754 ± 1.589	10.005 ± 0.648	2136.437 ± 99.780	267.845 ± 19.743	5.267 ± 0.481	88.466 ± 6.948	38.712 ± 2.135	4730.12 ± 242.662
C18	0.020 ± 0.001	2.387 ± 0.037	67.864 ± 4.545	3.973 ± 0.231	561.720 ± 14.421	214.872 ± 11.329	53.869 ± 2.864	261.25 ± 19.852	9.295 ± 0.167	174.037 ± 16.834	51.528 ± 3.689	2035.213 ± 180.924	264.218 ± 24.368	143.639 ± 10.622	94.697 ± 4.688	42.077 ± 3.269	3980.66 ± 297.841
C19	0.005 ± 0	0.045 ± 0.001	0.005 ± 0	ND	284.729 ± 12.468	25.108 ± 1.357	20.01 ± 1.302	82.787 ± 6.324	6.618 ± 0.325	31.781 ± 2.568	10.005 ± 0.586	516.409 ± 26.950	39.639 ± 3.800	53.17 ± 4.680	30.171 ± 2.220	12.294 ± 0.850	1082.775 ± 63.431
C20	0.030 ± 0.001	0.747 ± 0.035	104.284 ± 8.632	0.040 ± 0.001	722.378 ± 40.453	253.744 ± 13.526	70.116 ± 4.250	29.11 ± 1.955	7.451 ± 0.620	30.671 ± 1.962	3.141 ± 0.214	1583.879 ± 99.581	307.147 ± 27.624	254.53 ± 18.220	20.102 ± 1.955	15.432 ± 1.025	3402.801 ± 220.054
C21	ND	0.882 ± 0.037	54.698 ± 4.684	0.030 ± 0.001	957.578 ± 67.358	303.110 ± 24.021	90.731 ± 6.352	76.017 ± 5.955	7.412 ± 0.524	54.865 ± 3.261	36.292 ± 2.980	2098.668 ± 132.050	387.223 ± 26.352	256.108 ± 18.650	25.545 ± 2.050	37.646 ± 2.522	4386.803 ± 296.777
C22	0.010 ± 0	0.039 ±	0.015 ± 0	0.185 ±	337.435 ±	121.936 ±	20.01 ±	53.766 ±	7.873 ±	78.308 ±	0.005 ± 0	1011.833 ±	265.806 ±	151.393 ±	100.367 ±	82.946 ±	2211.926±1

		0.001		0.002	21.274	9.064	1.362	6.344	0.512	5.322		80.320	18.623	10.321	8.200	5.325	66.67
C23	0.020 ± 0	0.030 ± 0	121.989 ± 8.642	0.040 ± 0.002	1554.385 ± 96.067	674.190 ± 46.521	176.517 ± 10.550	21.106 ± 1.842	7.662 ± 0.600	35.445 ± 2.662	11.412 ± 0.950	1987.503 ± 150.320	456.953 ± 30.250	336.667 ± 23.120	18.753 ± 1.232	15.448 ± 1.203	5418.119 ± 373.961
C24	0.030 ± 0.001	1.138 ± 0.009	5.652 ± 0.067	15.565 ± 0.557	3282.203 ± 100.278	574.655 ± 42.147	19.608 ± 1.025	194.09 ± 12.300	32.359 ± 1.652	78.568 ± 5.300	2.387 ± 0.165	2056.994 ± 162.300	300.832 ± 16.952	29.788 ± 1.865	201.102 ± 18.230	49.625 ± 3.562	6934.595 ± 366.41
C25	ND	0.025 ± 0.001	59.034 ± 0.485	0.010 ± 0	1981.281 ± 75.658	729.938 ± 50.847	108.621 ± 9.627	27.527 ± 2.031	6.468 ± 0.538	60.372 ± 4.327	1.412 ± 0.102	1919.628 ± 150.348	592.72 ± 41.850	168.517 ± 10.584	144.018 ± 10.000	144.751 ± 9.581	5944.32 ± 365.979
C26	0.025 ± 0	4.322 ± 0.147	0.020 ± 0	7.904 ± 0.480	905.701 ± 80.084	179.494 ± 12.251	0.015 ± 0	193.873 ± 12.560	11.589 ± 1.075	115.65 ± 9.375	0.005 ± 0	3021.041 ± 200.242	306.569 ± 27.310	51.077 ± 4.235	159.945 ± 12.842	37.94 ± 2.534	4995.17 ± 363.135
C27	0.002 ± 0	0.060 ± 0.005	0.015 ± 0	0.015 ± 0.001	1788.048 ± 97.657	271.449± 14.481	0.015 ± 0.001	104.718 ± 7.562	6.518 ± 0.620	30.405 ± 1.530	0.005 ± 0	1657.84 ± 99.356	160.145 ± 10.578	51.746 ± 3.124	101.894 ± 7.625	23.656 ± 1.350	4196.531 ± 243.89
C28	0.030 ± 0.001	3.585 ± 0.127	0.025 ± 0.001	0.030 ± 0.002	969.609 ± 90.574	167.595 ± 10.432	0.02 ± 0.001	99.073 ± 7.357	6.468 ± 0.430	31.575 ± 2.220	0.005 ± 0	2008.362 ± 120.368	137.776 ± 10.248	50.151 ± 3.625	42.561 ± 3.165	14.271 ± 1.320	3531.135 ± 249.871
C29	0.015 ± 0.001	7.182 ± 0.653	8.505 ± 0.713	7.306 ± 0.625	1189.846 ± 94.418	309.591 ± 20.546	61.788 ± 4.369	112.968 ± 10.262	15.109 ± 1.048	58.237 ± 4.832	4.919 ± 0.251	1925.764 ± 142.362	202.513 ± 19.280	105.265 ± 9.649	97.129 ± 7.752	31.281 ± 2.163	4137.421 ± 318.924
C30	0.023 ± 0.001	0.941 ± 0.021	51.165 ± 3.426	7.716 ± 0.537	718.219 ± 35.542	217.672 ± 12.359	50.55 ± 4.025	116.282 ± 8.361	14.55 ± 1.038	67.128 ± 4.589	22.169 ± 1.953	2217.339 ± 176.340	398.482 ± 28.634	166.109 ± 12.366	110.669 ± 9.628	52.115 ± 4.325	4211.129 ± 303.145
C31	0.025 ± 0	3.216 ± 0.084	116.169 ± 8.954	1.981 ± 0.025	1114.743 ± 87.578	318.502 ± 27.230	152.551 ± 12.300	552.254 ± 46.320	7.046 ± 0.452	419.532 ± 30.247	108.137 ± 9.350	3001.359 ± 200.310	582.735 ± 49.652	406.583 ± 26.970	124.475 ± 10.958	102.1 ± 9.625	7011.406 ± 520.055
C32	0.025 ± 0	2.351 ± 0.018	0.015 ± 0	0.020 ± 0	1086.846 ± 80.157	123.999 ± 8.358	0.015 ± 0.001	256.134 ± 20.348	6.468 ± 0.560	179.583 ± 16.327	0.01 ± 0	1703.402 ± 142.387	167.176 ± 13.621	107.79 ± 9.628	204.223 ± 19.637	35.968 ± 2.480	3874.026 ± 313.522
C33	0.010 ± 0	0.045 ± 0	16.388 ± 0.103	0.015 ± 0	671.871 ± 15.035	168.957 ± 9.148	134.607 ± 10.584	111.378 ± 10.275	7.734 ± 0.380	90.642 ± 7.600	28.327 ± 2.231	1141.408 ± 97.652	210.793 ± 19.357	142.241 ± 11.284	50.591 ± 4.169	39.323 ± 2.853	2814.329 ± 190.671
C34	0.035 ± 0.001	0.885 ± 0.008	0.045 ± 0.001	0.015 ± 0	1232.545 ± 94.513	298.360 ± 10.249	1.373 ± 0.083	168.324 ± 12.274	6.468 ± 0.321	78.287 ± 5.948	0.015 ± 0.001	1634.071 ± 142.950	231.019 ± 20.510	11.538 ± 1.087	143.007 ± 10.598	34.691 ± 2.649	3840.68 ± 301.193

C35	0.020 ± 0.001	0.030 ± 0.001	87.732 ± 3.598	0.020 ± 0.001	968.389 ± 26.685	299.374 ± 9.652	45.602 ± 3.865	30.65 ± 2.951	6.468 ± 0.420	19.604 ± 1.262	1.241 ± 0.130	1303.82 ± 99.351	262.385 ± 22.563	156.887 ± 11.648	38.706 ± 2.955	21.996 ± 1.874	3242.925± 186.957
C36	0.030 ± 0.001	0.045 ± 0.001	39.768 ± 3.534	0.040 ± 0.001	1196.082 ± 30.533	121.925 ± 10.674	34.745 ± 2.959	208.914 ± 17.485	6.79 ± 0.621	81.083 ± 6.582	16.837 ± 1.104	2436.61 ± 177.350	236.445 ± 18.573	165.968 ± 14.381	119.627 ± 10.478	29.238 ± 2.452	4694.146± 296.729
C37	0.005 ± 0	0.020 ± 0	16.132 ± 0.942	0.045 ± 0.001	366.940 ± 26.696	184.435 ± 12.340	178.722 ± 12.658	29.014 ± 2.035	8.601 ± 0.730	35.125 ± 2.958	57.903 ± 4.628	641.319 ± 43.687	165.06 ± 12.365	160.997 ± 10.548	24.506 ± 1.985	85.473 ± 7.364	1954.297± 138.937
C38	0.005 ± 0	0.015 ± 0.001	21.091 ± 1.890	2.166 ± 0.128	1058.006 ± 88.245	406.359 ± 30.261	117.87 ± 10.274	5.837 ± 0.453	9.884 ± 0.805	9.633 ± 0.830	0.065 ± 0.005	1384.841 ± 110.340	288.276 ± 21.330	110.65 ± 9.658	20.446 ± 2.006	21.463 ± 1.995	3456.607± 278.221
C39	0.005 ± 0	0.020 ± 0.001	8.160 ± 0.607	0.015 ± 0	198.195 ± 16.958	93.281 ± 8.570	48.661 ± 3.998	42.002 ± 3.957	6.468 ± 0.452	63.108 ± 5.724	6.435 ± 0.353	876.316 ± 68.572	342.895 ± 29.675	158.188 ± 12.359	20.652 ± 1.958	34.952 ± 2.968	1899.353± 156.152
C40	0.040 ± 0.002	5.482 ± 0.452	192.091 ± 10.278	2.970 ± 0.240	1189.071 ± 100.201	352.682 ± 30.358	194.279 ± 15.627	130.591 ± 11.524	9.534 ± 0.857	126.03 ± 10.658	36.256 ± 3.251	4059.643 ± 312.600	837.463 ± 59.625	603.73 ± 52.344	88.333 ±5.366	58.255 ± 4.825	7886.449± 618.208
C41	0.060 ± 0.003	10.561 ± 0.980	81.193 ± 5.357	1.038 ± 0.023	2045.516 ± 105.320	407.807 ± 34.025	110.481 ± 9.958	98.905 ± 8.351	7.335 ± 0.652	56.167 ± 4.362	6.033 ± 0.515	4097.962 ± 265.321	522.112 ± 42.352	289.609 ± 20.349	141.308 ± 10.358	39.362 ± 2.899	7915.451± 510.824
C42	0.020 ± 0.001	0.040 ± 0.002	58.867 ± 4.195	2.814 ± 0.135	1326.997 ± 90.350	725.651 ± 60.866	72.31 ± 5.350	20.095 ± 1.974	12.516 ± 1.035	30.814 ± 2.367	0.045 ± 0.001	1804.32 ± 98.336	490.879 ± 32.020	103.611 ± 8.314	46.426 ± 3.985	55.461 ± 4.218	4750.867± 313.179
C43	0.030 ± 0.001	0.747 ± 0.030	0.010 ± 0.001	0.025 ± 0.001	1674.308 ± 100.310	191.411 ± 10.561	0.02 ± 0.001	96.362 ± 7.310	6.468 ± 0.527	29.355 ± 2.101	0.005 ± 0	2987.142 ± 198.477	208.244 ± 18.580	150.032 ± 10.890	153.492 ± 10.692	27.694 ± 2.108	5525.345± 361.59
C44	0.015 ± 0.001	0.020 ± 0.001	29.188 ± 2.028	0.015 ± 0.001	1569.667 ± 120.289	267.191 ± 20.290	156.236 ± 10.674	7.534 ± 0.627	13.618 ± 0.968	4.592 ± 0.540	4.77 ± 0.355	1370.879 ± 58.352	212.398 ± 30.589	205.435 ± 5.628	18.586 ± 1.035	10.694 ± 0.364	3870.839± 251.742
C45	0.010 ± 0	0.045 ± 0.001	0.005 ± 0	0.010 ± 0.001	513.660 ± 31.230	93.598 ± 4.251	0.005 ± 0	158.886 ± 7.537	6.468 ± 0.528	90.553 ± 6.359	0.005 ± 0	1038.618 ± 67.0	142.69 ± 8.360	166.66 ± 10.232	122.681 ± 6.384	30.77 ± 1.658	2364.664± 143.800
C46	0.010 ± 0	0.025 ± 0.001	41.894 ± 3.135	0.010 ± 0.001	599.375 ± 45.267	312.458 ± 25.191	156.34 ± 10.320	54.305 ± 3.266	7.462 ± 0.652	44.821 ± 3.694	21.457 ± 1.870	870.239 ± 60.210	302.056 ± 15.623	255.924 ± 15.678	28.178 ± 2.684	75.646 ± 6.523	2770.2± 194.115
C47	0.015 ± 0.001	0.065 ±	11.138 ±	6.524 ±	1091.406 ±	514.684 ±	24.759 ±	9.996 ±	15.609 ±	10.928 ±	0.03 ± 0.001	1575.637 ±	264.846 ±	46.004 ±	22.979 ±	20.28 ±	3614.9±

		0.003	1.028	0.350	90.982	33.015	1.865	0.873	1.068	0.960		100.265	23.477	3.523	1.958	1.527	260.896
C48	0.005 ± 0	0.015 ± 0	83.271 ± 6.528	ND	472.892 ± 35.416	314.009 ± 23.660	19.252 ± 1.233	1.031 ± 0.095	6.901 ± 0.433	4.169 ± 0.359	0.025 ± 0	1257.676 ± 99.368	384.252 ± 20.457	119.373 ± 9.368	18.008 ± 0.838	30.343 ± 2.695	2711.224± 200.45
C49	0.005 ± 0	0.025 ± 0	0.020 ± 0	0.030 ± 0.001	351.764 ± 31.560	205.936 ± 10.330	0.025 ± 0.001	12.447 ± 0.986	8.045 ± 0.754	20.074 ± 1.865	0.01 ± 0	869.724 ± 46.892	157.002 ± 10.356	150.221 ± 13.260	31.42 ± 2.356	24.151 ± 2.110	1830.899± 120.47
C50	0.020 ± 0.001	0.070 ± 0.004	45.591 ± 3.256	0.025 ± 0.001	1491.704 ± 101.035	214.709 ± 18.138	204.545 ± 19.764	151.498 ± 13.230	6.968 ± 0.582	32.471 ± 3.018	26.627 ± 1.856	2524.8 ± 100.230	288.354 ± 20.351	316.547 ± 28.310	127.213 ± 11.470	70.5 ± 6.890	5501.642± 328.134
C51	0.005 ± 0	0.015 ± 0	6.147 ± 0.500	0.030 ± 0.001	565.875 ± 50.000	158.186 ± 12.223	47.48 ± 3.522	17.744 ± 1.203	8.284 ± 0.750	10.01 ± 0.561	0.065 ± 0.003	889.761 ± 70.263	120.332 ± 9.354	58.79 ± 4.367	28.099 ± 1.958	23.256 ± 1.869	1934.079± 156.564
C52	0.200 ± 0.010	0.030 ± 0.002	124.252 ± 4.359	ND	313.490 ± 17.560	304.070 ± 18.365	185.399 ± 11.563	29.657 ± 2.355	9.884 ± 3.500	67.444 ± 4.356	37.811 ± 2.560	1071.994 ± 78.652	419.656 ± 28.353	402.265 ± 29.357	21.318 ± 1.364	98.54 ± 7.648	3105.81± 209.004
C53	0.010 ± 0	0.020 ± 0.001	0.005 ± 0	0.015 ± 0	532.009 ± 44.859	13.905 ± 1.234	50.015 ± 3.862	38.658 ± 2.550	6.468 ± 0.523	2.952 ± 0.195	0.01 ± 0	547.986 ± 40.360	217.398 ± 18.362	315.245 ± 25.600	38.834 ± 2.577	9.834 ± 0.485	1773.364± 140.608
C54	0.010 ± 0.001	0.045 ± 0.002	17.719 ± 1.213	0.045 ± 0.003	680.497 ± 48.890	324.098 ± 26.883	43.798 ± 3.250	15.154 ± 1.023	8.534 ± 0.652	10.323 ± 0.840	2.639 ± 0.135	2041.117 ± 153.230	334.714 ± 27.871	119.082 ± 9.658	30.548 ± 2.590	20.335 ± 1.362	3648.66± 277.603
C55	0.020 ± 0.001	3.141 ± 0.120	41.635 ± 2.354	0.725 ± 0.032	1170.604 ± 88.251	300.808 ± 22.235	230.87 ± 22.478	152.359 ± 11.325	9.212 ± 0.742	120.107 ± 10.250	63.801 ± 5.237	1879.776 ± 125.398	391.771 ± 26.350	314.254 ± 28.672	79.114 ± 6.386	119.082 ± 9.367	4877.279± 359.798
C56	0.020 ± 0.009	0.075 ± 0.005	0.055 ± 0.004	0.005 ± 0	1034.801 ± 79.365	131.914 ± 9.239	0.206 ± 0.009	188.615 ± 10.342	7.446 ± 0.354	90.521 ± 7.366	0.01 ± 0	1945.935 ± 133.262	203.929 ± 18.678	56.599 ± 4.689	88.905 ± 7.361	29.41 ± 2.136	3778.445± 272.819
C57	0.020 ± 0.001	0.045 ± 0.002	0.035 ± 0.001	0.100 ± 0.009	573.136 ± 44.300	72.926 ± 5.000	0.188 ± 0.009	162.604 ± 13.302	7.051 ± 0.512	57.476 ± 4.388	0.01 ± 0	1408.821 ± 86.350	122.709 ± 10.200	53.075 ± 4.986	80.652 ± 7.958	21.918 ± 1.867	2560.766± 172.885
C58	0.030 ± 0.001	4.546 ± 0.350	81.389 ± 6.568	5.791 ± 0.350	1594.089 ± 124.540	683.959 ± 57.338	70.016 ± 5.688	29.181 ± 1.987	11.422 ± 0.852	50.945 ± 4.352	0.117 ± 0.010	3101.4 ± 253.200	576.592 ± 48.362	215.01 ± 18.600	23.04 ± 1.958	25.678 ± 2.165	6473.204± 520.411
C59	0.010 ± 0	0.025 ± 0.001	41.941 ± 2.791	0.010 ± 0	599.254 ± 44.384	312.956 ± 24.000	156.261 ± 12.330	54.378 ± 4.627	7.451 ± 0.562	44.865 ± 3.955	21.298 ± 1.847	872.745 ± 75.685	303.021 ± 24.623	255.972 ± 20.300	28.46 ± 1.580	75.26 ± 4.369	2773.907± 221.099

C60	0.015 ± 0	2.892 ± 0.154	0.010 ± 0	0.075 ± 0.005	753.541 ± 66.340	153.586 ± 13.295	0.025 ± 0.001	104.86 ± 9.658	8.462 ± 0.652	111.616 ± 10.368	0.005 ± 0	1868.191 ± 155.330	173.74 ± 10.677	261.68 ± 23.644	57.488 ± 4.852	24.811 ± 1.780	3520.998± 296.756
C61	0.015 ± 0.001	0.569 ± 0.040	0.010 ± 0.001	1.099 ± 0.650	640.470 ± 42.340	198.291 ± 17.250	0.005 ± 0	64.477 ± 5.362	8.584 ± 0.458	41.653 ± 2.740	0.005 ± 0	1618.572 ± 99.326	191.978 ± 10.339	265.595 ± 24.331	37.768 ± 2.867	22.09 ± 1.955	3091.181± 207.66
C62	0.010 ± 0.001	0.055 ± 0.002	22.027 ± 2.011	0.010 ± 0	559.571 ± 48.361	159.480 ± 10.360	131.929 ± 9.352	37.035 ± 1.687	6.862 ± 0.523	36.843 ± 2.895	7.979 ± 0.652	1053.623 ± 85.352	291.814 ± 15.628	218.428 ± 14.357	22.907 ± 1.250	24.612 ± 1.957	2573.185± 194.379
C63	0.010 ± 0	0.055 ± 0.002	51.119 ± 4.032	0.020 ± 0.001	790.132 ± 45.300	317.759 ± 17.214	85.974 ± 5.310	32.052 ± 2.144	6.946 ± 0.530	28.672 ± 2.034	34.378 ± 2.854	1266.501 ± 84.320	295.357 ± 15.377	220.981 ± 18.352	20.047 ± 1.857	28.943 ± 2.180	3178.946± 201.507
C64	0.010 ± 0	0.200 ± 0	34.940 ± 2.523	0.015 ± 0.001	440.901 ± 33.250	113.377 ± 10.033	53.012 ± 4.820	40.934 ± 3.265	6.546 ± 0.567	19.487 ± 1.560	5.129 ± 0.421	1015.209 ± 81.366	150.837 ± 13.624	91.549 ± 5.688	23.118 ± 1.958	14.854 ± 1.687	2010.119± 161.083
C65	0.035 ± 0.001	0.829 ± 0.071	0.030 ± 0.001	43.762 ± 3.239	1645.055 ± 120.055	101.933 ± 9.358	0.065 ± 0.004	271.182 ± 20.544	33.197 ± 2.980	30.892 ± 2.652	0.01 ± 0	2231.48 ± 200.327	69.917 ± 5.320	52.722 ± 4.700	145.773 ± 12.580	16.642 ± 1.230	4643.524± 383.062
C66	0.200 ± 0.009	0.050 ± 0.005	2.532 ± 0.108	27.584 ± 1.250	1552.197 ± 122.321	320.633 ± 25.770	8.601 ± 0.530	28.402 ± 1.881	13.577 ± 1.247	24.891 ± 1.965	0.05 ± 0.001	2328.933 ± 189.560	296.456 ± 25.347	30.379 ± 2.560	154.464 ± 12.320	58.227 ± 4.652	4866.976± 399.526
C67	0.030 ± 0.001	0.065 ± 0.002	69.272 ± 4.051	0.035 ± 0.001	1653.831 ± 120.000	599.869 ± 48.036	87.088 ± 4.652	179.647 ± 14.660	6.724 ± 0.524	136.621 ± 11.544	20.067 ± 1.842	2339.085 ± 199.350	590.104 ± 47.326	213.021 ± 17.369	106.259 ± 9.650	95.586 ± 7.689	6097.304± 486.697
C68	0.025 ± 0.001	0.734 ± 0.035	104.345 ± 7.630	0.040 ± 0.001	722.196 ± 62.363	254.838 ± 22.345	70.277 ± 4.357	29.132 ± 1.953	7.18 ± 0.548	30.685 ± 2.960	3.131 ± 0.125	1583.925 ± 124.352	307.324 ± 27.625	254.52 ± 21.352	20.124 ± 1.892	15.316 ± 1.240	3403.79± 166.687
C69	0.020 ± 0.001	0.030 ± 0.001	11.896 ± 0.957	22.265 ± 1.358	1436.031 ± 100.560	421.410 ± 22.068	25.666 ± 2.135	48.248 ± 3.945	16.054 ± 1.240	21.522 ± 1.855	0.045 ± 0.001	1918.073 ± 123.550	218.707 ± 20.354	42.467 ± 3.560	34.724 ± 2.663	18.153 ± 1.253	4235.311± 285.51
C70	0.100 ± 0.009	0.040 ± 0.002	16.360 ± 0.930	0.065 ± 0.003	641.672 ± 55.038	229.963 ± 20.410	20.142 ± 1.855	30.732 ± 2.688	9.339 ± 0.652	23.144 ± 1.958	1.17 ± 0.104	1218.207 ± 99.675	238.694 ± 20.662	67.54 ± 4.852	38.24 ± 2.743	33.836 ± 2.631	2579.143± 214.212
C71	0.015 ± 0.001	0.055 ± 0.002	0.005 ± 0	0.005 ± 0	740.535 ± 60.320	88.425 ± 7.055	0.005 ± 0	144.952 ± 12.440	6.468 ± 0.426	36.061 ± 2.477	0.005 ± 0	1120.576 ± 100.366	41.966 ± 3.557	53.501 ± 4.200	72.633 ± 4.531	15.52 ± 1.241	2320.728± 196.616
C72	0.015 ± 0.001	0.277 ±	8.512 ±	0.030 ±	833.479 ±	159.093 ±	11.696 ±	203.499 ±	8.206 ±	102.634 ±	4.400 ±	2431.718 ±	289.731 ±	61.842 ±	69.229 ±	29.099 ±	4213.459±

		0.015	0.347	0.001	66.245	12.245	0.985	18.330	0.752	9.600	0.300	189.668	25.340	5.324	5.847	1.956	336.956
C73	0.015 ± 0.001	0.040 ± 0.002	27.523 ± 1.900	0.045 ± 0.002	420.620 ± 30.038	110.229 ± 10.500	17.409 ± 1.274	109.528 ± 9.367	9.956 ± 0.590	60.749 ± 5.332	13.112 ± 1.102	1375.849 ± 100.241	234.501 ± 22.387	94.782 ± 8.670	45.037 ± 3.600	21.707 ± 1.871	2541.103± 196.877
C74	0.005 ± 0	0.005 ± 0	10.650 ± 0.990	0.015 ± 0.001	406.942 ± 30.660	156.795 ± 10.358	58.341 ± 4.688	79.948 ± 6.952	6.99 ± 0.562	84.174 ± 6.355	12.568 ± 1.022	948.128 ± 84.365	248.207 ± 21.368	125.517 ± 10.376	32.692 ± 2.958	51.007 ± 4.251	2221.985± 184.906
C75	0.005 ± 0	0.030 ± 0.001	4.934 ± 0.300	0.075 ± 0.003	536.957 ± 34.162	157.574 ± 13.365	32.276 ± 2.957	43.741 ± 3.958	8.856 ± 0.968	48.814 ± 3.600	1.59 ± 0.120	721.789 ± 53.218	137.476 ± 11.785	33.695 ± 2.931	21.613 ± 1.501	23.617 ± 1.952	1773.042± 130.821
C76	0.030 ± 0.001	0.055 ± 0.001	0.023 ± 0.001	12.783 ± 0.940	1024.762 ± 97.037	385.851 ± 17.034	1.899 ± 0.123	164.86 ± 15.690	9.569 ± 0.856	147.177 ± 13.289	2.516 ± 0.154	1551.577 ± 100.037	259.313 ± 21.228	51.512 ± 4.099	60.665 ± 6.200	22.368 ± 1.978	3694.96± 278.668
C77	0.020 ± 0.001	0.070 ± 0.004	0.025 ± 0.001	0.035 ± 0.002	1366.128 ± 127.000	151.807 ± 10.038	0.21 ± 0.014	90.112 ± 7.528	6.74 ± 0.547	56.494 ± 4.948	0.005 ± 0	1414.707 ± 121.385	108.264 ± 9.664	50.508 ± 3.501	102.633 ± 9.229	33.514 ± 2.459	3381.273± 296.321
C78	0.055 ± 0.004	12.817 ± 1.033	250.944 ± 21.300	7.975 ± 0.644	709.960 ± 55.374	242.314 ± 21.340	188.747 ± 16.860	242.684 ± 22.697	7.379 ± 0.632	157.773 ± 13.250	95.544 ± 7.920	2739.694 ± 222.380	503.014 ± 34.665	667.38 ± 48.313	158.668 ± 12.877	146.545 ± 12.613	6141.491± 491.902
C79	0.015 ± 0.001	2.458 ± 0.197	8.374 ± 0.663	1.177 ± 0.098	1348.857 ± 126.848	250.965 ± 26.569	98.87 ± 8.000	192.93 ± 15.188	9.778 ± 0.860	81.417 ± 6.958	18.832 ± 1.574	1839.496 ± 156.340	227.537 ± 20.681	99.946 ± 7.958	121.42 ± 10.352	58.638 ± 4.220	4360.712± 386.607
C80	0.015 ± 0.001	0.025 ± 0.001	23.517 ± 1.891	1.611 ± 0.120	1429.144 ± 122.430	382.322 ± 34.035	41.255 ± 3.928	90.393 ± 8.379	8.645 ± 0.652	45.111 ± 2.498	2.476 ± 0.153	1566.491 ± 135.000	225.194 ± 19.669	62.918 ± 4.958	44.282 ± 3.697	27.971 ± 1.948	3951.368± 339.360
C81	0.020 ± 0.001	0.035 ± 0.002	15.094 ± 1.329	0.030 ± 0.001	1330.612 ± 111.740	185.271 ± 14.684	15.673 ± 1.233	186.83 ± 14.417	6.874 ± 0.562	77.771 ± 4.380	13.664 ± 0.957	1555.417 ± 108.297	181.093 ± 17.518	73.22 ± 5.270	48.497 ± 3.462	17.542 ± 1.300	3707.642± 285.117
C82	0.015 ± 0.001	0.045 ± 0.003	0.020 ± 0	11.849 ± 0.950	1830.871 ± 139.870	549.742 ± 49.773	2.088 ± 0.135	40.561 ± 2.620	9.001 ± 0.752	34.243 ± 2.621	0.01 ± 0.000	1378.982 ± 121.194	320.131 ± 28.622	56.46 ± 4.957	49.869 ± 3.491	22.951 ± 1.344	4306.846± 356.333
C83	0.020 ± 0	0.040 ± 0.002	0.010 ± 0.001	11.625 ± 0.995	839.082 ± 81.353	67.988 ± 4.564	0.035 ± 0.002	161.433 ± 13.620	14.51 ± 1.300	14.08 ± 1.021	0.045 ± 0.001	1030.204 ± 96.342	36.696 ± 2.867	51.134 ± 4.500	54.406 ± 3.840	11.822 ± 0.953	2293.13± 211.359
C84	0.005 ± 0	0.015 ± 0.001	7.001 ± 0.095	5.798 ± 0.245	673.980 ± 45.351	259.197 ± 20.038	6.759 ± 0.482	11.067 ± 0.966	10.289 ± 0.384	18.662 ± 1.155	0.03 ± 0	727.11 ± 56.460	148.827 ± 12.697	66.575 ± 4.680	18.358 ± 1.327	18.281 ± 1.050	1971.952± 144.901

C85	0.020 ± 0.001	6.695 ± 0.455	33.670 ± 2.332	22.095 ± 2.039	1258.489 ± 114.580	368.298 ± 30.806	52.041 ± 3.733	177.402 ± 14.890	13.327 ± 0.953	107.578 ± 9.687	19.91 ± 1.380	1875.927 ± 160.890	350.081 ± 28.600	125.336 ± 11.956	98.223 ± 9.313	35.569 ± 2.822	4544.662± 394.437
C86	0.030 ± 0.001	0.192 ± 0.068	0.010 ± 0.001	3.647 ± 0.175	2952.324 ± 200.045	751.855 ± 38.762	0.015 ± 0.001	98.621 ± 8.698	6.64 ± 0.547	45.993 ± 3.130	0.005 ± 0	2195.193 ± 168.360	390.139 ± 29.697	54.568 ± 4.623	72.15 ± 3.528	23.206 ± 1.986	6594.587± 459.622
C87	0.010 ± 0	0.045 ± 0.002	8.978 ± 0.710	26.370 ± 2.351	951.651 ± 84.037	185.463 ± 16.750	3.002 ± 0.280	102.733 ± 9.682	13.965 ± 1.035	33.386 ± 2.541	1.54 ± 0.103	1128.473 ± 95.362	130.306 ± 11.240	25.792 ± 1.852	83.795 ± 7.623	29.91 ± 2.130	2725.422± 235.668
C88	0.020 ± 0.001	0.025 ± 0.001	12.401 ± 1.020	0.015 ± 0.001	1532.984 ± 143.000	87.810 ± 7.026	26.851 ± 2.362	84.84 ± 7.658	6.907 ± 0.560	11.113 ± 0.945	3.002 ± 0.210	1316.021 ± 112.643	84.29 ± 6.304	55.142 ± 4.952	61.931 ± 4.382	15.165 ± 1.243	3298.516± 292.308
C89	0.010 ± 0.001	0.035 ± 0.002	0.030 ± 0.001	0.010 ± 0.001	246.188 ± 22.350	47.348 ± 2.761	0.03 ± 0.001	134.116 ± 10.414	6.563 ± 0.307	60.721 ± 4.021	0.005 ± 0	712.643 ± 58.670	75.165 ± 5.950	125.438 ± 10.333	36.735 ± 3.186	15.115 ± 1.240	1460.151± 119.22
C90	0.010 ± 0	ND	0.010 ± 0	0.020 ± 0.001	568.824 ± 51.670	121.502 ± 10.078	0.005 ± 0	125.788 ± 10.984	7.079 ± 0.587	91.901 ± 8.625	0.005 ± 0	1084.023 ± 95.675	127.185 ± 11.895	158.347 ± 13.151	18.819 ± 1.275	14.41 ± 1.110	2317.928± 205.651
C91	0.150 ± 0.009	0.055 ± 0.003	0.030 ± 0.001	12.788 ± 1.009	1022.521 ± 89.290	385.819 ± 2.860	1.974 ± 0.164	164.592 ± 14.616	9.500± 0.657	147.175 ± 11.263	2.651 ± 0.135	1556.305 ± 130.250	314.339 ± 28.681	56.55 ± 4.852	60.665 ± 5.687	22.368 ± 1.958	3772.333± 291.435
C92	0.010 ± 0.001	0.015 ± 0.001	0.015 ± 0.001	9.316 ± 0.840	964.529 ± 89.344	317.613 ± 24.088	0.02 ± 0	7.588 ± 0.531	8.09 ± 0.250	14.126 ± 1.152	ND	787.199 ± 56.892	142.002 ± 12.358	104.568 ± 8.695	53.956 ± 4.692	30.715 ± 2.877	2439.761± 177.634
C93	0.150 ± 0.011	0.025 ± 0.002	11.536 ± 0.996	0.020 ± 0.001	1408.593 ± 135.000	183.621 ± 14.035	89.973 ± 7.358	27.11 ± 2.342	6.551 ± 0.053	3.617 ± 0.023	2.17 ± 0.013	1530.582 ± 126.300	160.867 ± 14.150	106.817 ± 9.680	25.117 ± 2.154	13.643 ± 1.147	3585.243± 313.264
C94	0.020 ± 0.001	0.020 ± 0.001	0.065 ± 0.003	0.015 ± 0.001	1568.906 ± 126.380	95.433 ± 8.125	0.055 ± 0.001	33.702 ± 3.120	6.835 ± 0.571	6.609 ± 0.552	0.02 ± 0	1012.122 ± 99.320	70.617 ± 6.124	79.598 ± 4.598	53.873 ± 4.315	17.414 ± 1.540	2945.304± 254.652
C95	0.015 ± 0.001	0.055 ± 0.003	104.156 ± 9.500	0.010 ± 0.001	464.963 ± 36.890	290.193 ± 21.067	268.009 ± 23.500	43.457 ± 3.210	6.468 ± 0.322	51.952 ± 3.268	10.981 ± 0.872	2055.911 ± 156.582	341.873 ± 28.817	260.78 ± 21.643	94.991 ± 8.117	107.464 ± 9.867	4101.278± 323.66
C96	0.005 ± 0	0.020 ± 0.001	0.060 ± 0.003	0.005 ± 0	357.683 ± 28.433	90.378 ± 7.110	28.9 ± 2.230	98.937 ± 8.795	6.851 ± 0.521	91.068 ± 8.715	7.687 ± 0.527	712.565 ± 60.200	141.88 ± 12.100	44.033 ± 2.850	22.318 ± 2.045	21.874 ± 1.856	1624.265± 135.386
C97	0.010 ± 0	0.035 ±	41.728 ±	ND	418.913 ±	167.940 ±	54.958 ±	15.841 ±	7.351 ±	32.77 ±	0.907 ±	1290.803 ±	350.098 ±	139.965 ±	20.052 ±	30.587 ±	2571.956±

		0.002	2.098		38.079	14.260	4.851	1.247	0.594	2.495	0.008	100.303	33.200	11.596	1.573	2.594	212.900
C98	0.010 ± 0	0.035 ± 0.001	0.010 ± 0	2.376 ± 0.180	1230.706 ± 100.350	313.355 ± 25.068	0.01 ± 0.001	31.358 ± 2.697	10.567 ± 0.680	24.912 ± 1.562	ND	1606.976 ± 130.240	239.005 ± 20.341	104.568 ± 9.653	87.472 ± 6.850	38.24 ± 2.412	3689.599± 300.035
C99	0.005 ± 0	0.015 ± 0.001	290.723 ± 23.560	0.020 ± 0.001	298.133 ± 24.030	288.407 ± 22.160	139.509 ± 11.852	3.806 ± 0.240	7.029 ± 0.489	10.771 ± 0.846	0.075 ± 0.005	649.588 ± 51.320	401.596 ± 31.588	175.869 ± 15.550	19.125 ± 1.432	66.807 ± 5.880	2351.477± 188.953

[a] The sample No. is same as Table S1.

[b] Concentrations of the target compounds were calculated from the corresponding standards using external standard method.

[c] Quantified relatively by calibration curves of maslinic acid.

[d] Quantified relatively by calibration curves of oleanolic acid.

[e] Quantified relatively by calibration curves of betulinic acid.

ND: not detected, Datas were showed as means ± SD (n = 3).

.

Table S3 Component matrixa of principal component analysis

Variables	Component			
	PC1	PC2	PC3	PC4
Maslinic acid isomer-1	0.215	0.173	0.033	0.347
Maslinic acid isomer-2	0.612	-0.061	0.279	0.068
Maslinic acid isomer-3	0.648	-0.452	-0.270	0.178
Maslinic acid isomer-4	0.102	0.602	0.143	0.688
Alphitolic acid	0.390	0.800	-0.120	-0.253
Maslinic acid	0.574	0.385	-0.618	-0.110
2α- hydroxy ursolic acid	0.595	-0.524	-0.295	0.135
Maslinic acid isomer-5	0.361	0.121	0.826	-0.116
Oleanolic acid isomer-1	0.118	0.611	0.081	0.634
Maslinic acid isome-6	0.510	-0.113	0.640	-0.177
Maslinic acid isomer-7	0.658	-0.485	0.292	0.128
Betulinic acid	0.721	0.343	0.195	-0.143
Oleanolic acid	0.831	-0.105	-0.308	-0.053
Ursolic acid	0.687	-0.580	-0.087	0.120
Betulonic acid	0.392	0.476	0.566	-0.151
Oleanonic acid + Ursonic acid	0.651	-0.296	0.093	0.035
Total triterpenic acids	0.412	0.720	-0.121	-0.237
FRAP	0.524	0.417	-0.616	-0.092
